# Prevalence of osteoarthritis in lower middle- and low-income countries: a systematic review and meta-analysis

**DOI:** 10.1007/s00296-021-04838-y

**Published:** 2021-04-27

**Authors:** Ismail Yahaya, Tanya Wright, Opeyemi O. Babatunde, Nadia Corp, Toby Helliwell, Lisa Dikomitis, Christian D. Mallen

**Affiliations:** 1 School of Medicine, Keele University, Keele, Staffordshire, Newcastle upon Tyne, ST5 5BG UK; 2 Institute for Global Health, Keele University, Keele, Staffordshire, Newcastle upon Tyne, ST5 5BG UK; 3 Research and Innovation Department, Midlands Partnership Foundation Trust, St George’s Hospital, Block 7, Corporation Street, Stafford, ST16 3AG UK

**Keywords:** Osteoarthritis, Prevalence, Low-income and lower middle-income countries

## Abstract

Evidence from the Global Burden of Disease studies suggests that osteoarthritis (OA) is a significant cause of disability globally; however, it is less clear how much of this burden exists in low-income and lower middle-income countries. This study aims to determine the prevalence of OA in people living in low-income and lower middle-income countries. Four electronic databases (MEDLINE, EMBASE, CINAHL and Web of Science) were systematically searched from inception to October 2018 for population-based studies. We included studies reporting the prevalence of OA among people aged 15 years and over in low-income and lower middle-income countries. The prevalence estimates were pooled across studies using random effects meta-analysis. Our study was registered with PROSPERO, number CRD42018112870.The search identified 7414 articles, of which 356 articles were selected for full text assessment. 34 studies were eligible and included in the systematic review and meta-analysis. The pooled prevalence of OA was 16·05% (95% confidence interval (CI) 12·55–19·89), with studies demonstrating a substantial degree of heterogeneity (*I*^2^ = 99·50%). The pooled prevalence of OA was 16.4% (CI 11·60–21.78%) in South Asia, 15.7% (CI 5·31–30·25%) in East Asia and Pacific, and 14.2% (CI 7·95–21·89%) in Sub Saharan Africa. The meta-regression analysis showed that publication year, study sample size, risk of bias score and country-income categories were significantly associated with the variations in the prevalence estimates. The prevalence of OA is high in low-income and lower middle-income countries, with almost one in six of the study participants reported to have OA. With the changing population demographics and the shift to the emergence of non-communicable diseases, targeted public health strategies are urgently needed to address this growing epidemic in the aging population.

**Supplementary Information:**

The online version contains supplementary material available at 10.1007/s00296-021-04838-y.

## Introduction

Low- and middle-income countries (LMICs) are experiencing a dramatic shift in the burden of disease from communicable to non-communicable disease (NCD) [1]. This is causing a significant challenge for governments and health care systems that are already strained due to the epidemics associated with HIV/AIDS, other infectious diseases and weak health systems. The prevalence of NCD continues to grow and was responsible for 70% of deaths worldwide in 2016 [2]. NCDs also accounted for 61% of global disability adjusted life years (DALYs) in 2016, around 20% higher than in 1990, [3] with the highest rise observed in LMICs settings. Musculoskeletal conditions account for a significant proportion of NCDs contributing to DALYs, with osteoarthritis (OA) contributing most to this burden. OA carries an excess mortality and financial burden both societally and to individuals suffering from it [4], and is a major contributor to the global disability burden, with an increase of 9·6% of the global age-standardised years lived with disability (YLD) between 1990 and 2017 [5]. The Global Burden of Disease (GBD) study (2015) ranked OA and diabetes highest in terms of largest increase in years lived with disability when compared to the other top causes of disability [6].

Osteoarthritis is an important public health issue and is the most common type of arthritis [7], with 10% of the world’s population aged 60 years and above having health problems attributed to OA [8]. It is clinically characterised by joint pain, stiffness and functional limitation. Osteoarthritis is common in the joints of the knees, hands, hips, and feet, while also affecting the joints of the shoulder and the spine. The exact cause of OA is unknown, but it is thought to be related to ageing, whilst also associated with modifiable (trauma, obesity, lack of exercise) and non-modifiable risk factors (gender, age, genetics) [9]. The economic cost associated with OA is enormous, ranging from direct treatment and care costs to lost work productivity [3, 10–12].

To date, the majority of research on musculoskeletal disorders has been conducted in high-income settings, with limited data only from LMICs despite findings from the GBD 2010 study suggesting that the prevalence of arthritis may be higher in LMICs [13]. Furthermore, where evidence from LMICs is available it is typically from the upper middle-income group without much evidence from the lower middle income and the low-income countries that make up the remaining LMICs bloc.

Existing evidence is generally country specific with disparate methodologies and estimates within regions. In the Africa region estimates vary between 4.88 and 36.8% [14, 15], 2.96% and 56.99% in East Asia Pacific [16, 17] and 1.42 and 83.73 in South Asia [18, 19]. The aim of this study, therefore, is to provide a robust evidence synthesis of data on the prevalence of OA in lower middle and low-income countries which has always been under-represented and will be a rising contributor to YLD. We aimed to pool data from population-based studies in different regions of low-income and lower middle-income countries to calculate contemporary prevalence estimates of OA.

## Methods

### Search strategy and selection criteria

We performed a systematic search in four electronic bibliographic databases: MEDLINE, EMBASE, CINAHL and Web of Science from database inception to October 2018. Terms (both subject headings and text words) were combined for osteoarthritis (including OA, arthrosis, degenerative, knee pain, hip pain, hand pain, joint pain, finger pain, thumb pain), prevalence [including incidence, occurrence, disease rate, disease frequency, disease pattern), and LMICs (including developing countries and specifically named countries) [see Supplementary table S1 for the search strategy]. It is important to note that our search strategy incorporated LMICs which comprises of low-income, lower middle and upper-middle-income countries, but it was at the selection stage that we restricted included studies to low-income and lower middle-income economies. Reference lists of eligible studies and review articles were also assessed.

We included population-based studies of adults and adolescents (aged 15 years or over) that reported prevalence estimates of OA in low-income and lower middle-income countries (see detail exclusion and inclusion criteria in Table 1). Studies were excluded if the study population were not based in a lower middle and low-income countries, if they included other types of arthritis such as rheumatoid arthritis or if they were editorials, expert reviews, commentaries and traditional reviews. Searches were limited to human studies only, without any other limitations applied including language and year of publication to maximise the opportunity for study inclusion. Low-income countries are defined as economies with Gross National Income (GNI) per capita, calculated using the World Bank Atlas method, of $995 or less in 2018, while lower middle-income countries are those with a GNI per capita of more than $996 but less than $3895 [20, 21].

**Table 1:** Study inclusion and exclusion criteria

	Inclusion	Exclusion
Population	Adults and adolescents (15 years and above +)	Adolescents and children under 15 years of age
Outcome	OA prevalence defined based on ACR definition	OA prevalence not reported
Study design	Population-based studies: cross-sectional studies	Hospital-based studies, reviews, policy report, other primary study designs i.e. not cross-sectional
Study location	Lower middle-income countries Low-income countries	High-income countries Upper-middle-income countries

Two reviewers (IY and TW) independently checked study eligibility. All the identified articles were initially screened by their titles and the resulting studies by abstract to determine potential eligibility. In the event of discrepancies, agreement was reached by consensus and by discussion with a third reviewer (TH). The full texts of potentially eligible studies were obtained and further assessed by the two reviewers (IY and TW) for final inclusion. At this stage, all upper middle-income countries were excluded, retaining only the low-income and lower middle-income countries.

Data extraction was undertaken by two reviewers independently (IY and TW) using a pre-designed extraction proforma. Data extracted included information on country and region of study, income category, study setting, type of study, sampling strategy, sample size, study design, mean age, diagnostic criteria for OA, site of OA, and effect estimates (prevalence). The countries were grouped by regions and income according to World Bank development indicators [21]. The total prevalence estimates were only calculated from studies that provided prevalence data.

Risk of bias was assessed by two reviewers independently (IY and TW) using an adapted version of the risk of bias tool for prevalence studies [22]. This is a validated and widely adopted measure used to assess bias in cross-sectional studies. The checklist consists of ten questions, with a maximal score of 1 for each question. A score of 1 (yes) or 0 (no) was assigned to each question, and scores summed across questions to generate an overall quality score that ranged from 0 to 10. An overall score of 0–4, 5–7 and 8–10 were used to classify the study as either high, moderate or low risk of bias. Any disagreement was resolved through consensus. The items for this tool are included in the electronic supplementary material. Given the limited published data on OA from LMICs we included conference abstracts within our analysis providing they had achieved the data requirements for the study.

### Data analysis

Meta-analysis was undertaken using the random effect model as there were anticipated variations in the study population and methodologies. Heterogeneity was assessed by inspecting the forest plots and using the Chi-squared test for heterogeneity with a 10% level of statistical significance, and using the *I*^2^ statistic, where we interpreted a value of 50% as representing moderate heterogeneity [23]. Publication bias was assessed by funnel plot asymmetry and Egger’s test for regression asymmetry [24]. We used the “trim and fill” analysis of Duval and Tweedie [25] to examine the potential impact of missed or unpublished studies on the pooled estimates of OA prevalence.

OA prevalence estimate was reported with 95% confidence interval (CI). Analyses were conducted using Stata Statistical Software release 15 (State Corp, college Station, TX) using the “metaprop” routine [26].

The systematic review rationale, objectives and protocol were pre-specified and published in PROSPERO register (CRD42018112870) [27] and reported according to preferred Reporting Items for Systematic Reviews and Meta-analysis (PRISMA) guidelines [28].

## Results

**Fig. 1:**
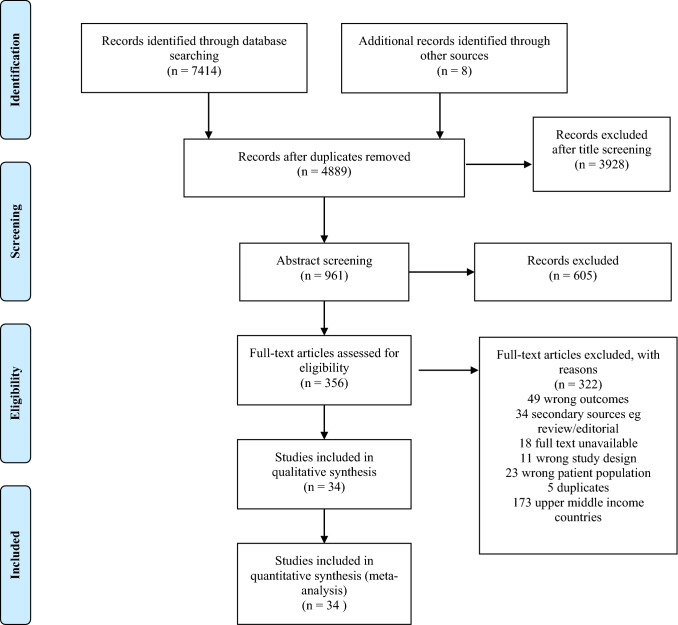
PRISMA flow diagram showing study selection

Figure 1 shows the study flowchart. We identified 7414 articles from the electronic search, of which 2525 were duplicates. After initial screening of abstract, 356 articles were selected for full text screening. Three hundred and twenty-two (322) studies were excluded for not meeting the selection criteria, leaving 34 studies to be included in the review. The reasons for exclusion are provided in Fig. 1.

Supplementary table S2 summarizes the characteristics of the included studies. In total, 80,000 people from 25 countries were included in this review. Most of the studies were conducted in India (*n* = 11) [18, 29–38] and Nigeria (*n* = 7) [29–45]. Three of the studies were conducted in each of Vietnam [16, 46, 47] and Bangladesh [48–50], two from Sri Lanka [51, 52] and Philippines [17, 53], while one study each from Indonesia [54], Democratic Republic of Congo [14], Pakistan [55], Burkina Faso [15], Ukraine [56] and Cameroon [57] (see included countries and regions in Table 2). More than half of the participants in the studies were female. Of the included studies, 27 (79%) were conducted between 2005 and 2017, with only seven (21%) studies being published before 2005.

**Table 2:** Regions and countries included in the study

Regions	Countries
East Asia and Pacific	Indonesia, Philippines, Vietnam
Europe and Central Asia	Ukraine
South Asia	Bangladesh, Pakistan, India, Sri Lanka
Sub-Saharan Africa	Nigeria, Democratic Republic of Congo, Burkina Faso, Cameroon

Osteoarthritis was defined using American College of Rheumatology (ACR) criteria or using self-reported physician diagnosis. ACR criteria was used in 38% [13] of the studies, while the remaining were based on self-reported physician/clinical diagnosis 12 (35%) or COPCORD based questionnaire eight (24%). In 50% (*n* = 17) of the included studies, the site of OA was the knee [17, 18, 33, 34, 37, 38, 42, 47–50, 54, 55], 38% (*n* = 13) was generalised OA [14, 15, 29–32, 35, 36, 44–46, 53, 57], while the remaining 11% (*n* = 4) of the studies were either hand OA [56], spine OA [47] or the site was not stated [39, 43].

Thirty one of the included studies used point prevalence as a measure while it was unclear what prevalence estimate was used in two [18, 35] of the included studies. Only one of the studies [39] used period prevalence as a measure of estimate. 94·1% (*n* = 32) of the studies included a mixed population, comprising of both males and females. Only 5·8% (*n* = 2) of the studies [37, 52] used male or female population.

The summary of the risk of bias assessment is shown in supplementary table S3. Of the 34 included studies, 11 (32·4%) had moderate risk of bias [15, 17, 30, 39, 42–45, 48, 56, 57], and four (11·8%) had high risk of bias [14, 18, 33, 35].

**Fig. 2:**
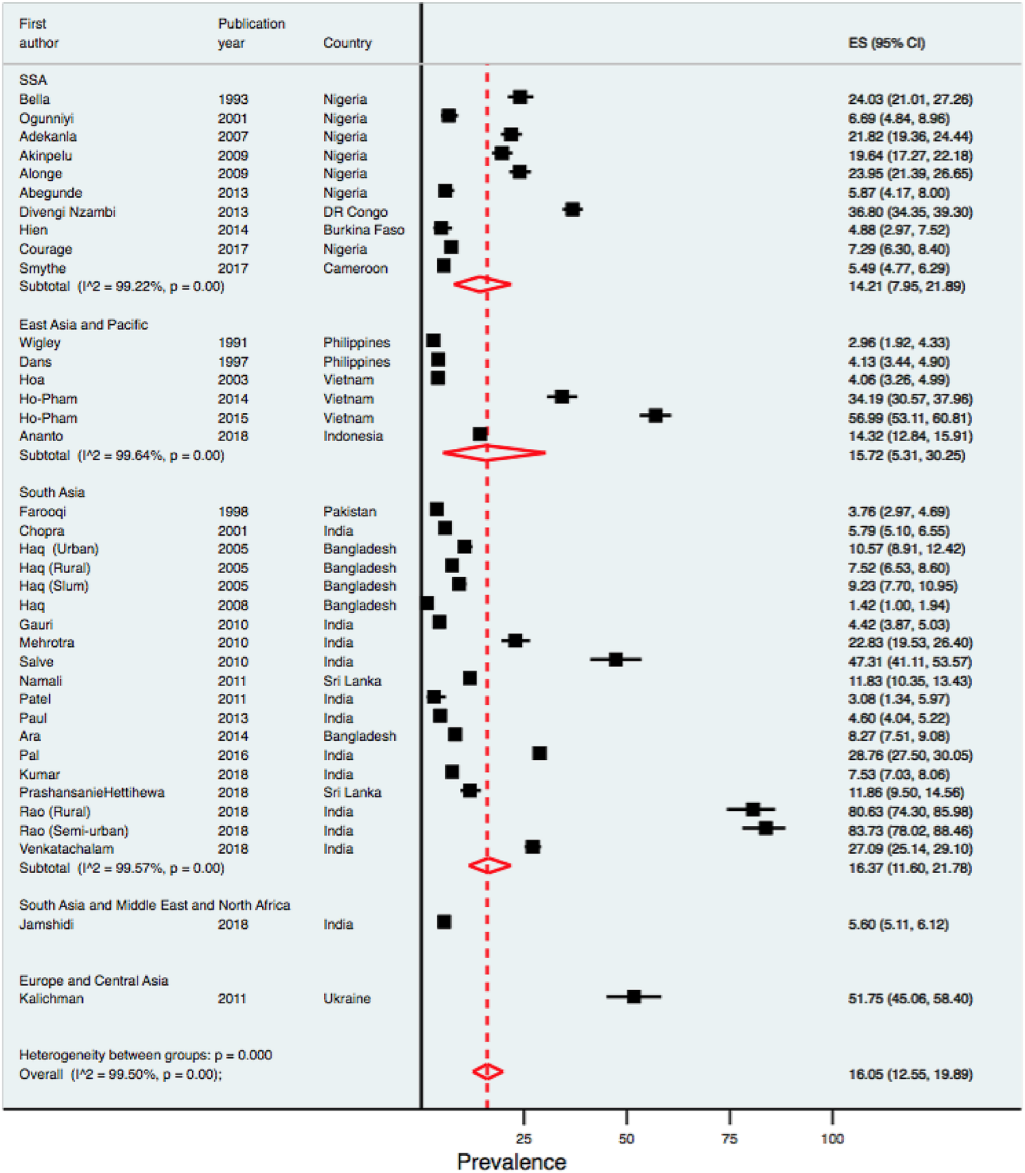
Forest plot showing the OA prevalence estimates by regions. *ES* Effect size, *CI* confidence interval

**Fig. 3:**
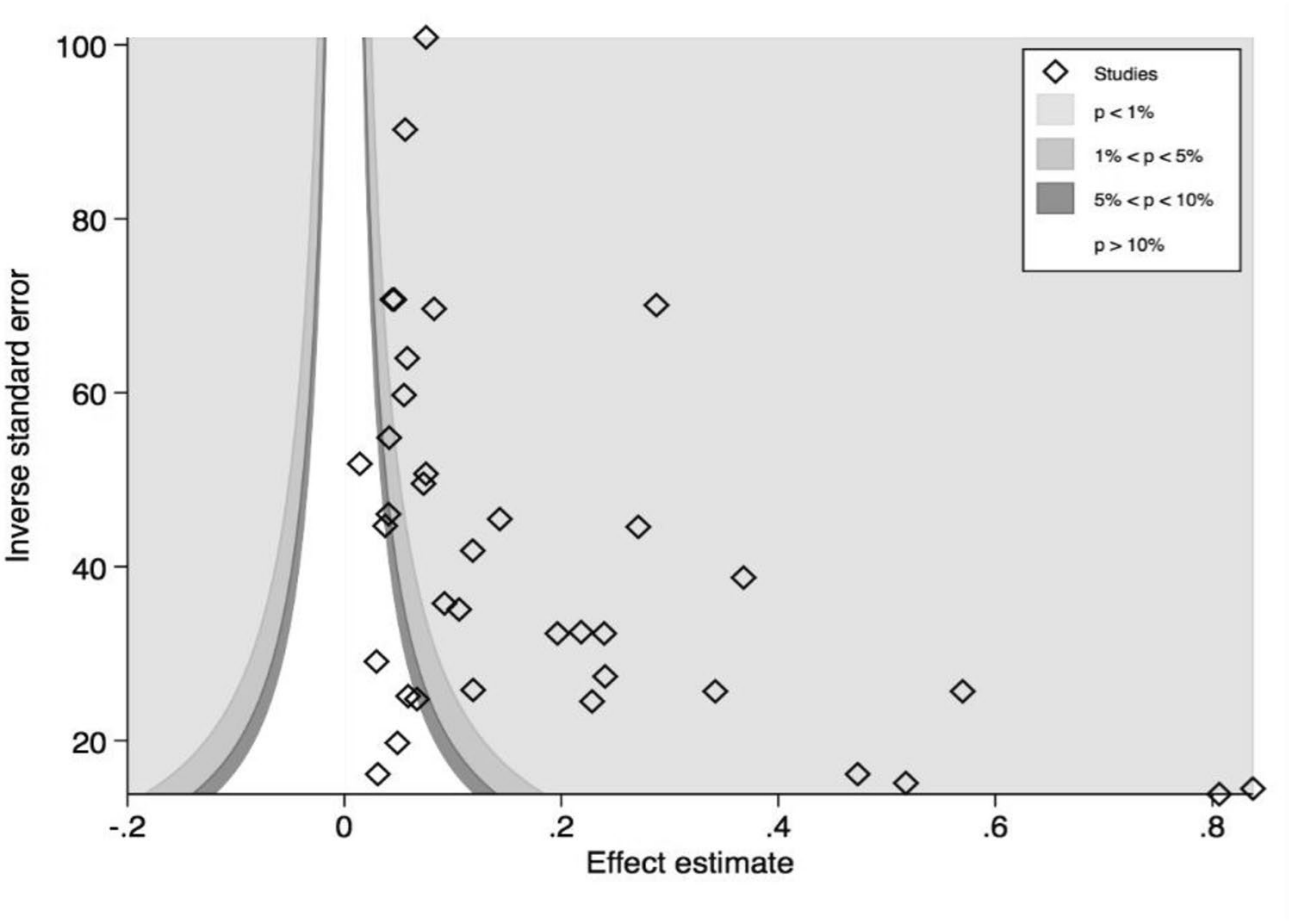
Funnel plot

The prevalence estimates of OA based on individual study/country is shown in Fig. 2. The prevalence of OA in this population varied widely across the countries. The prevalence varied from 5·49% (95% CI 4.77–6.29) to 36·80% (95% CI 34.35–39.30) in Sub-Saharan Africa (SSA), from 2·96% (1.92–4.33) to 56·99% (53.11–60.11) in East Asia and Pacific (EAP), from 1·42% (1.00–1.94) to 83·73% (78.02–88.46) in South Asia (SA). The reported prevalence of OA ranges from 1.42 (1.00–1.94) [19] to 83.73 (78.02–88.46) [18]. The pooled OA prevalence from all included studies was 16.05 (12.55–19.89). The *I*^2^ was 99·50% indicating a high level of heterogeneity across the studies. The funnel plot for assessing publication bias (Fig. 3) was asymmetrical, indicating a potential for publication bias. This was also confirmed by Egger’s test (*p* value = 0·001) for small-study effect.

**Fig. 4:**
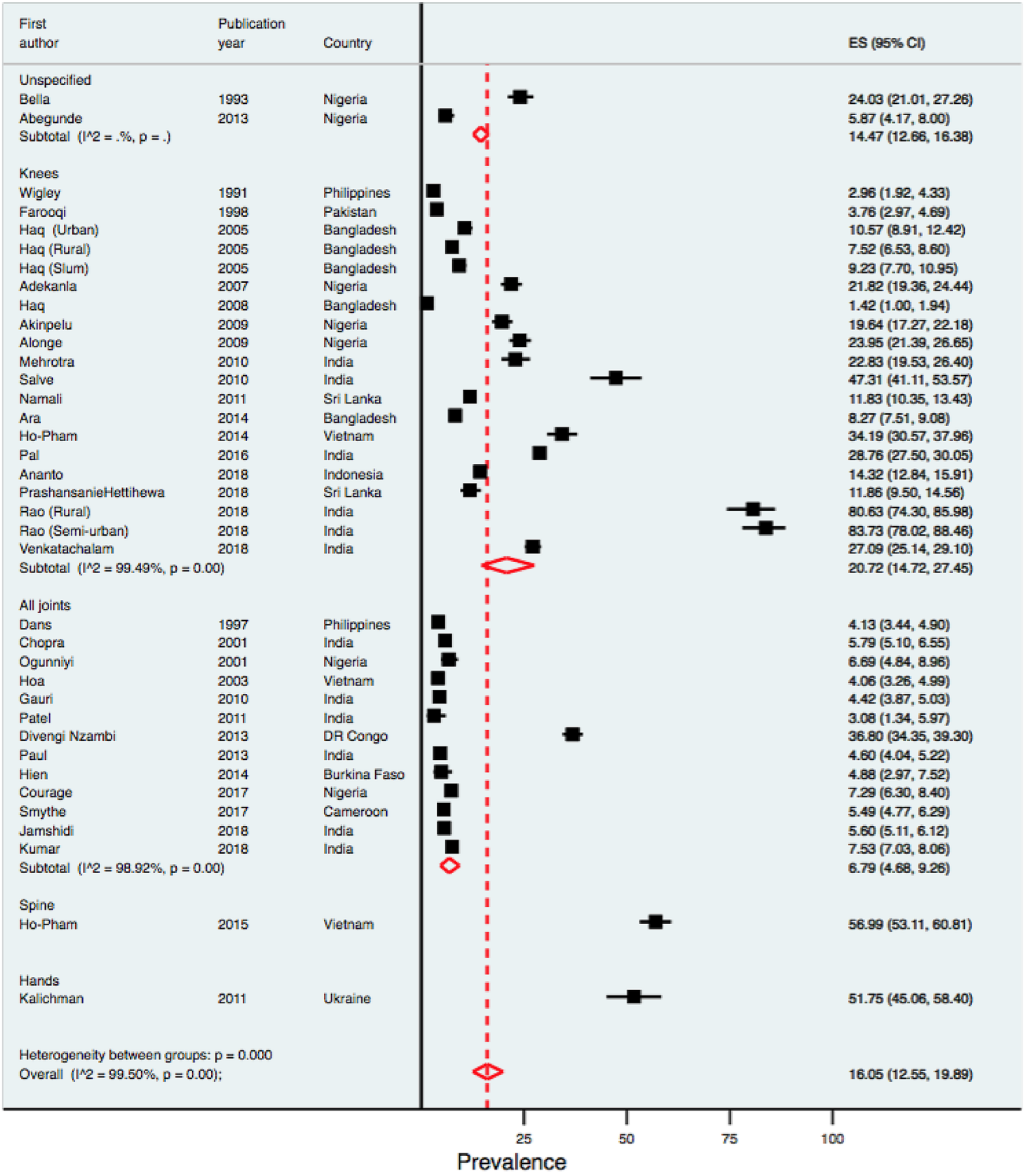
Forest plot showing the OA prevalence estimates by sites of OA. *ES* Effect size, *CI* confidence interval

**Fig. 5:**
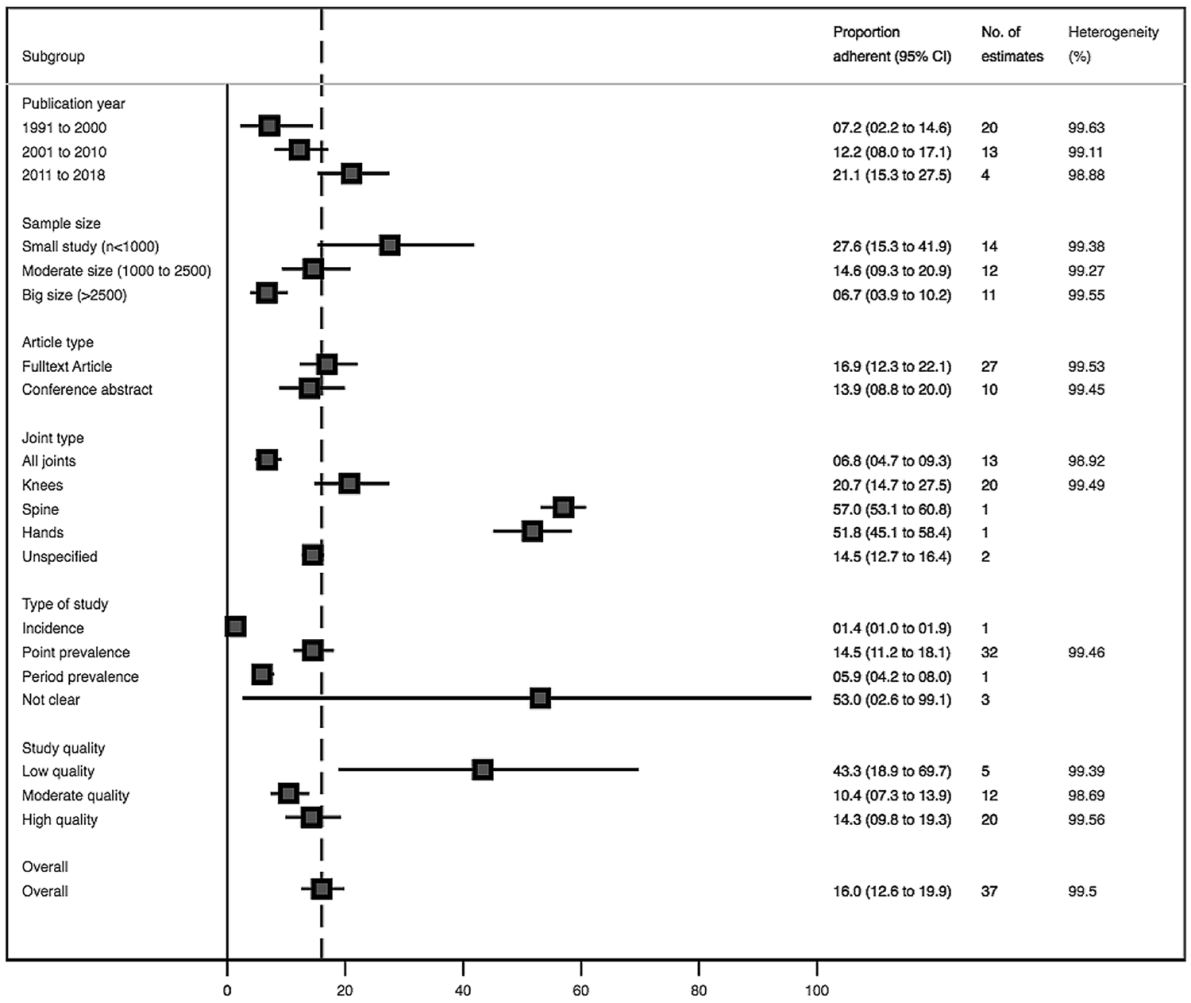
Pooled prevalence estimates by different subgroups

The results of the sub-group analysis are shown in Figs. 2, 4, 5. The knee is the prevalent site of OA, with a reported prevalence of 20·72% (14.72–27.45). There was no evidence of differential burden of OA across different sub-regions except for one outlier study each from South Asia and Europe/Central Asia (Fig. 2). The reported prevalence estimates were higher among studies with small samples than those from moderate and large sample sizes (27.8% vs 14.60% vs 6.72%). Similarly, the reported prevalence estimates tended to be higher among low quality studies than those reported by moderate and high-quality studies.

Secular trend in OA prevalence is shown in (Supplementary figure S1). We observed a continuous increasing trend in the prevalence of OA. The studies in the 90’s recorded a low prevalence ranging from 2·96 [17] to 3·7% [55], while the more recent studies showed an increasing trend with the most recent between 2010 and 2017 recording a prevalence as high as 57% [47].

The results of the study-level factors associated with the variations in the OA prevalence estimates are shown in Table 3. The results of the meta-regression analysis showed that publication year, study sample size, quality score and country-income categories were significantly associated with the variations in the prevalence estimates. For every 10-year increase in the study publication, the prevalence of OA increased by 10% (OR = 1.10, 95% CI 1.00–1.20) (Supplementary figure S1). As the study sample sizes (Supplementary figure S2) and study quality score (Supplementary figure S3) increases, the prevalence estimates reduces by 3% and 4%, respectively. The prevalence of OA tended to increase as the percentage of females included in the study increases (Supplementary figure S4). Country-income category (23.7%) explained the highest between studies variance, followed by OA sites (19.3%) and study sample size (13.6%).

**Table 3:** Factors associated with prevalence estimates

Factors	OR (95% CI)	% Variance explained
Publication year (per 10 years)	1.10 (1.00–1.20)	9.10
Sample size (per 1000)	0.97 (0.94–0.99)	13.62
Study quality score	0.96 (0.93–1.00)	9.95
Percent female	1.01 (1.00–1.01)	7.03

*OR* Odds ratio

## Discussion

This systematic review of the prevalence of OA in lower middle- and low-income countries has brought together evidence from 34 cross-sectional studies from the last 25 years, incorporating 80,000 participants. These studies were unevenly distributed with Nigeria and India, both lower middle income countries, accounting for about a third of the included studies. It is one of the first systematic reviews undertaken investigating the prevalence of OA specifically in lower middle- and low-income countries. This review highlights that OA is an important public health problem in these countries with 1 in 6 persons affected by the condition. This reinforces the fact that OA is highly prevalent, irrespective of setting, making it a major public health problem in the world that hitherto has rarely been considered. The prevalence estimates observed are comparable with the studies conducted in other regions of the world [58–60]. For example, using data from Framingham study, Kim and colleagues [58] estimated that the prevalence of OA in adults aged 50 years was 19.6%.

Our findings suggest that there is an increasing trend in the prevalence of OA over time, with more recent studies recording higher prevalence of the condition. The exact reason for the reported high prevalence or the increasing trend in the prevalence of OA in the lower middle- income and low-income countries is likely to be multifactorial. The reported rising trend in the burden of disease due to OA may reflect increasing life expectancy, and prolonged exposure to arthritis risk factors such as obesity, occupational factors, joint overuse, mechanical injury, genetics and gender [59]. Although the prevalence of OA increases with age, it can occur at any age, affecting people’s ability to work and hence personal earnings, activities of daily living, therefore, impacting on overall personal and societal productivity. For LMICs, the burden does not stop there, but further worsens the vicious cycle of disease and poverty [61]. There is also an established link between lower educational attainment, lower skilled job [62] and many chronic diseases including OA. The link could be explained by those with lower educational attainment having lower paid jobs of which most are long-term manual jobs and may involve heavy lifting and squatting [63, 64] and which for many LMICs represent a significant source of GDP income. This is not to imply that poverty in LMICs is a cause for OA, but rather, its impact more significant and potential for prevention and or treatment less in places in the world where healthcare is limited or unaffordable.

The overall quality of the included studies was moderate with more than half of the studies assessed as having a low risk of bias and about a third of them assessed as having a moderate risk of bias. This is a strength of this study as it makes it highly unlikely that sampling bias has impacted the overall prevalence of OA. Another strength lies in the comprehensive searches that were conducted to ensure that all relevant studies/publications were identified.

Potential bias was also reduced while conducting this review by ensuring that the different steps of the review from screening of the abstracts to the data extraction stage were independently carried out. There was reasonable coverage of evidence in three of the geographical regions (SSA, SA and EAP), which make up the vast majority of the low-middle income countries. This allows for generalisability of the results across these regions. However, there is limited data to provide evidence for other regions like Latin America and the Caribbean.

Several limitations have to be acknowledged. The high degree of heterogeneity across included studies was a major limitation in this study. The heterogeneity could be explained by the differences in population characteristics and study methodologies. The heterogeneity observed between the studies may have been as a result of cultural or geographical differences as well as methodological differences employed in the studies. Despite the heterogeneity, it was still observed that the pooled estimate for each of the sub regions was still modest, especially in the three sub-regions of SSA, EAP and SA with pooled estimates of 14·21 (7·95–21·89), 15·72 (5·31–30·25) and 16·37 (11·60–21·78), respectively.

A further limitation relates to generalisability. While there were primary studies of reasonable quality within some of the sub-regions, studies from Nigeria, India and Bangladesh predominated, making the results unlikely to be generalisable. We also found some evidence of publication bias in this review. However, studies have shown that testing for publication bias in the presence of significant heterogeneity may lead to false-positive result [65]. Despite our ability to estimate the prevalence of OA, we were unable to examine the potential impact of different factors or potential correlates of OA including obesity and socioeconomic status which may have a role in predicting the distribution of OA. We can infer from the risk of bias result that the prevalence estimate from this review may not fulfil the rule of generalisability because the included studies were not representative of the national population.

In conclusion, based on available evidence, we found a high level of OA in low and lower middle income countries. There is a need for development of public health strategies for prevention and early management. In addition, future studies should examine the impact of OA on individuals and society as a whole in these settings.

## Supplementary Information

Below is the link to the electronic supplementary material.

Supplementary file1 (DOCX 358 KB)

Supplementary file2 Supplementary table S2 SSA Sub-Saharan Africa, LMI Lower and middle-income countries, ACR American College of Rheumatology, KL Kellgren Lawrence , XR X-ray (DOCX 26 KB)

Supplementary file3 (DOCX 40 KB)

## Abbreviations

OA: Osteoarthritis
CI: Confidence interval
LMIC: Low- and middle-income countries
NCD: Non-communicable disease
DALY: Disability adjusted life years
YLD: Years lived with disability
GBD: Burden of disease
GNI: Gross national income
ACR: American College of Rheumatology
COPCORD: Community oriented program for control of rheumatic diseases
SSA: Sub-Saharan Africa
EAP: East Asia and Pacific
SA: South Asia

## References

[1] Organization WH (2010). Global status report on noncommunicable diseases.

[2] Global Health Observatory data: Deaths from NCDs [Internet]. WHO. 2016 [cited 19 Novemeber 2019]. Available from: https://www.who.int/gho/ncd/mortality_morbidity/ncd_total/en/.

[3] DALYs GBD, Collaborators H (2017). Global, regional, and national disability-adjusted life-years (DALYs) for 333 diseases and injuries and healthy life expectancy (HALE) for 195 countries and territories, 1990–2016: a systematic analysis for the Global Burden of Disease Study 2016. Lancet (London, England).

[4] Palazzo C, Nguyen C, Lefevre-Colau M-M (2016). Risk factors and burden of osteoarthritis. Ann Phys Rehabil Med.

[5] Safiri S, Kolahi A-A, Smith E (2020). Global, regional and national burden of osteoarthritis 1990–2017: a systematic analysis of the Global Burden of Disease Study 2017. Ann Rheum Dis.

[6] Collaborators. GDaIIaP (2016). Global, regional, and national incidence, prevalence, and years lived with disability for 310 diseases and injuries, 1990–2015: a systematic analysis for the Global Burden of Disease Study 2015. Lancet.

[7] Picavet HS, Hazes JM (2003). Prevalence of self reported musculoskeletal diseases is high. Ann Rheum Dis.

[8] Woolf AD, Pfleger B (2003). Burden of major musculoskeletal conditions. Bull World Health Organ.

[9] Haq I, Murphy E, Dacre J (2003). Osteoarthritis. Postgrad Med J.

[10] Cross M, Smith E, Hoy D (2014). The global burden of hip and knee osteoarthritis: estimates from the global burden of disease 2010 study. Ann Rheum Dis.

[11] Chaganti RK, Lane NE (2011). Risk factors for incident osteoarthritis of the hip and knee. Curr Rev Musculoskelet Med.

[12] Altman RD (2010). Early management of osteoarthritis. Am J Manag Care.

[13] Storheim K, Zwart JA (2014). Musculoskeletal disorders and the Global Burden of Disease study. Ann Rheum Dis.

[14] Divengi Nzambi JP, Lukusa Mbaya A, Mulenge A et al. (2013) Prevalence of musculoskeletal disorders in a rural area of the Democratic Republic of Congo (DRC)2013; clinical rheumatology. Conference: 7th congress of the african league of associations for rheumatology, AFLAR and 23rd congress of the South African rheumatism and arthritis association, SARAA. Durban South Africa. Conference Publication: (var.pagings). 32 (2 SUPPL. 1) (pp S149); Springer London

[15] Hien H, Berthé A, Drabo MK (2014). Prevalence and patterns of multimorbidity among the elderly in Burkina Faso: cross-sectional study. Trop Med Int Health.

[16] Ho-Pham LT, Lai TQ, Mai LD (2015). Prevalence and pattern of radiographic intervertebral disc degeneration in Vietnamese: a population-based study. Calcif Tissue Int.

[17] Wigley R, Manahan L, Muirden KD (1991). Rheumatic disease in a Philippine village II: A WHO-ILAR-APLAR COPCORD study, phases II and III. Rheumatol Int.

[18] Rao BS, Ravindranath VS, Moorthy GVS, Prasad T (2018). Preservance of functional capacity in osteoarthritis knee in rural and semi-urban population. J Evol Med Dent Sci.

[19] Haq SA, Davatchi F, Dahaghin S (2010). Development of a questionnaire for identification of the risk factors for osteoarthritis of the knees in developing countries. A pilot study in Iran and Bangladesh. An ILAR-COPCORD phase III study. Int J Rheum Dis.

[20] New country classification by income level: 2018–2019 [Internet]. 2018 [cited November 2018]. Available from: https://blogs.worldbank.org/opendata/new-country-classifications-income-level-2018-2019.

[21] World Bank Country and Lending Groups [Internet]. World bank. 2018 [cited 2018]. Available from: https://datahelpdesk.worldbank.org/knowledgebase/articles/906519.

[22] Hoy D, Brooks P, Woolf A (2012). Assessing risk of bias in prevalence studies: modification of an existing tool and evidence of interrater agreement. J Clin Epidemiol.

[23] Higgins JP, Thompson SG (2002). Quantifying heterogeneity in a meta-analysis. Stat Med.

[24] Egger M, Davey Smith G, Schneider M, Minder C (1997). Bias in meta-analysis detected by a simple, graphical test. BMJ.

[25] Duval S, Tweedie R (2000). A non-parametric “Trim and Fill” method of accounting for publication bias in meta-analysis. J Am Stat Ass.

[26] Nyaga VN, Arbyn M, Aerts M (2014). PMC4373114; Metaprop: a Stata command to perform meta-analysis of binomial data. Arch Public Health.

[27] Yahaya IHT, Wright T, Babatunde O, Corp N, Mallen C (2018) Prevalence of osteoarthritis in low- and middle-income countries: a systematic review and meta-analysis. PROSPERO 10.1007/s00296-021-04838-y PMC8164595 33907879

[28] Moher D, Fau-Tetzlaff LA, J, Tetzlaff J Fau, Altman DG, Altman DG, (2009). Preferred reporting items for systematic reviews and meta-analyses: the PRISMA statement. PLoS Med.

[29] Chopra A, Patil J, Billempelly V, Relwani J, Tandle HS (2001). Prevalence of rheumatic diseases in a rural population in western India: a WHO-ILAR COPCORD Study. J Assoc Phys India.

[30] Gauri LA, Ranwa BL, Fatima Q, et al. (2010) Prevalence of rheumatic diseases in urban Bikaner population of Western Rajasthan: a who-ilar copcord study2010; Indian journal of rheumatology. Conference: annual conference of indian rheumatology association 2010, IRACON-2010. Bhubaneswar India. Conference publication: (var.pagings). 5 (3 SUPPL. 1) (pp S20); Elsevier (Singapore) Pte Ltd.

[31] Jamshidi A, Kianifard T, Ghorpade R, et al. (2018) Disparity in osteoarthritis knee prevalencea tale of two cities in Iran (Tehran) and India (Pune): findings from who ilar copcord population survey (stage i)2018 2018; Annals of the Rheumatic Diseases. Conference: Annual European Congress of Rheumatology, EULAR. Netherlands. 77 (Supplement 2) (pp 546); BMJ Publishing Group

[32] Kumar P, Alok R, Das SK, Srivastava R, Agarwal GG (2018). Distribution of rheumatological diseases in rural and urban areas: an adapted COPCORD Stage I Phase III survey of Lucknow district in north India. Int J Rheum Dis.

[33] Mehrotra A, Ozair M, Mehrotra R, et al. (2010) Self reported prevalence of rheumatological disease in a weaver dominated area in Varanasi2010; Indian journal of rheumatology. conference: annual conference of Indian Rheumatology Association 2010, IRACON-2010. Bhubaneswar India. Conference Publication: (var.pagings). 5 (3 SUPPL. 1) (pp S19-S20); Elsevier (Singapore) Pte Ltd

[34] Pal CP, Singh P, Chaturvedi S, Pruthi KK, Vij A (2016). Epidemiology of knee osteoarthritis in India and related factors. Indian J Orthop.

[35] Patel MM (2011). An epidemiological survey of arthritis in the population of north Gujarat, India. Int J Pharm Sci Res.

[36] Paul BJ, Rahim AA, Bina T, Thekkekara RJ (2013). Prevalence and factors related to rheumatic musculoskeletal disorders in rural south India: WHO-ILAR-COPCORD-BJD India Calicut study. Int J Rheum Dis.

[37] Salve H, Gupta V, Palanivel C, Yadav K, Singh B (2010). Prevalence of knee osteoarthritis amongst perimenopausal women in an urban resettlement colony in South Delhi. Indian J Public Health.

[38] Venkatachalam J, Natesan M, Eswaran M (2018). Prevalence of osteoarthritis of knee joint among adult population in a rural area of Kanchipuram District, Tamil Nadu. Indian J Public Health.

[39] Abegunde KA, Owoaje ET (2013). Health problems and associated risk factors in selected urban and rural elderly population groups of South-West Nigeria. Ann Afr Med.

[40] Adekanla BA, Alonge TO, Akinpelu AO (2007). Epidemiological profile of symptomatic knee osteoarthritis in adults: a population based study in Igbo-Ora. Nigeria Osteoporos Int.

[41] Akinpelu AO, Alonge TO, Adekanla BA, Odole AC (2009). Prevalence and pattern of symptomatic knee osteoarthritis in Nigeria: a community-based study. Internet J Allied Health Sci Pract.

[42] Alonge TO, Babatunde AO, Aderonke AO et al. (2009). Relationship between obesity and knee osteoarthritis in Nigeria2009; Osteoarthritis and Cartilage. Conference: 2009 World Congress on Osteoarthritis. Montreal, QC Canada. Conference Publication: (var.pagings). 17 (SUPPL. 1) (pp S169); W.B. Saunders Ltd

[43] Bella AF, Baiyewu O, Bamigboye A (1993). The pattern of medical illness in a community of elderly Nigerians. Cent Afr J Med.

[44] Courage UU, Stephen DP, Lucius IC (2017). Prevalence of musculoskeletal diseases in a semi-urban Nigerian community: results of a cross-sectional survey using COPCORD methodology. Clin Rheumatol.

[45] Ogunniyi A, Baiyewu O, Gureje O (2001). Morbidity pattern in a sample of elderly Nigerians resident in Idikan community. Ibadan West Afr J Med.

[46] Hoa TTM, Damarwan J, Le CS (2003). Prevalence of the rheumatic diseases in urban Vietnam: A WHO-ILAR COPCORD study. J Rheumatol.

[47] Ho-Pham LT, Lai TQ, Mai LD (2014). Prevalence of radiographic osteoarthritis of the knee and its relationship to self-reported pain. PLoS ONE.

[48] Ara R, Haq S, Hoy D (2014). Estimation of the prevalence of knee osteoarthritis and its impacts on quality of life in rural Bangladesh. Internet Med J.

[49] Haq SA, Darmawan J, Islam MN (2005). Prevalence of rheumatic diseases and associated outcomes in rural and urban communities in Bangladesh: a COPCORD study. J Rheumatol.

[50] Haq SA, Darmawan J, Islam N (2008). Incidence of musculoskeletal pain and rheumatic disorders in a Bangladeshi rural community: a WHO-APLAR-COPCORD study. Int J Rheum Dis.

[51] Namali H, Fonseka P, Gunathilake N, editors (2011). Prevalence of knee osteoarthritis among community dwelling older adults in Colombo district2011; Internal Medicine Journal. conference: 52nd annual scientific meeting of the australian rheumatology association in conjunction with rheumatology health professionals association. Brisbane, QLD Australia. Conference Publication: (var.pagings). 41 (SUPPL. 1) (pp 24); Blackwell Publishing

[52] Prashansanie Hettihewa A, Gunawardena NS, Atukorala I (2018). Prevalence of knee osteoarthritis in a suburban, Srilankan, adult female population: a population-based study. Int J Rheum Dis.

[53] Dans LF, TankerTorres S, Amante CM, Penserga EG (1997). The prevalence of rheumatic diseases in a Filipino urban population: a WHO-ILAR COPCORD study. J Rheumatol.

[54] Ananto M, Rahman PA, Al Rasyid H, et al., editors (2018). Risk factors for knee osteoarthritis in Malang population2018; International Journal of Rheumatic Diseases. Conference: 20th Asia Pacific League of Associations for Rheumatology Congress, APLAR 2018. Taiwan (Republic of China). 21 (Supplement 1) (pp 26); Blackwell Publishing

[55] Farooqi A, Gibson T (1998). Prevalence of the major rheumatic disorders in the adult population of north Pakistan. Br J Rheumatol.

[56] Kalichman L, Korosteshevsky M, Batsevich V, Kobyliansky E (2011). Climate is associated with prevalence and severity of radiographic hand osteoarthritis. Homo.

[57] Smythe T, Mactaggart I, Kuper H (2017). Prevalence and causes of musculoskeletal impairment in Fundong District, North-West Cameroon: results of a population-based survey. Trop Med Int Health.

[58] Kim C, Linsenmeyer KD, Vlad SC (2014). Prevalence of radiographic and symptomatic hip osteoarthritis in an urban United States community: the Framingham osteoarthritis study. Arthritis Rheumatol.

[59] Neogi T, Zhang Y (2013). Epidemiology of osteoarthritis. Rheum Dis Clin North Am.

[60] Turkiewicz A, Gerhardsson de Verdier M, Engstrom G (2015). Prevalence of knee pain and knee OA in southern Sweden and the proportion that seeks medical care. Rheumatology (Oxford).

[61] Nations U (2011). Prevention and control of non-communicable diseases: report of the secretary-general.

[62] Hannan MT, Anderson JJ, Pincus T, Felson DT (1992). Educational attainment and osteoarthritis: differential associations with radiographic changes and symptom reporting. J Clin Epidemiol.

[63] Andersen S, Thygesen LC, Davidsen M, Helweg-Larsen K (2012). Cumulative years in occupation and the risk of hip or knee osteoarthritis in men and women: a register-based follow-up study. Occup Environ Med.

[64] Kirkhorn S, Greenlee RT, Reeser JC (2003). The epidemiology of agriculture-related osteoarthritis and its impact on occupational disability. WMJ.

[65] Higgins JP (2008). Commentary: heterogeneity in meta-analysis should be expected and appropriately quantified. Int J Epidemiol.

