# Supplementary material for: Prevalence of osteoarthritis in lower middle- and low-income countries: a systematic review and meta-analysis
Source: Rheumatol Int. 2021 Apr 27;41(7):1221–31. doi: 10.1007/s00296-021-04838-y (PMC8164595; doi:10.1007/s00296-021-04838-y)
Supplement: Supplementary file 1 — Supplementary file1 (DOCX 358 KB) [file 296_2021_4838_MOESM2_ESM.docx]

**SUPPLEMENTARY TABLE**

**Supplementary table S2: Summary study characteristics**

| **Study** | **Country** | **Region** | **Income** | **Publication** | **Site** | **Measure** | **Age** | **Gender** | **Female** | **Outcome** |
| --- | --- | --- | --- | --- | --- | --- | --- | --- | --- | --- |
| Abegunde, 2013 | Nigeria | SSA | LMI | Journal | Unspecified | Clinical - history plus exam evidence of joint deformity or crepitus | 60-110 | Male and female | 61·1 | period prevalence |
| Adekanla, 2007 | Nigeria | SSA | LMI | Conference abstract | Knees | Clinical diagnosis - based on ACR criteria | 40+ | Male and female |  | point prevalence |
| Akinpelu, 2009 | Nigeria | SSA | LMI | Journal | Knees | Clinical - based on ACR criteria with smaller random sample of radiographs to confirm | 40+ | Male and Female | 53 | point prevalence |
| Alonge, 2009 | Nigeria | SSA | LMI | Conference abstract | Knees | Clinical - ACR criteria | 40+ | Male and female | 53 | point prevalence |
| Ananto, 2018 | Indonesia | East Asia and Pacific | LMI | Conference abstract | Knees | Interview and COPCORD questionnaire | 15+ | Male and female |  | point prevalence |
| Ara, 2014 | Bangladesh | South Asia | LMI | Conference abstract | Knees | Interview and COPCORD methodology | 15+ | Male and female |  | point prevalence |
| Bella, 1993 | Nigeria | SSA | LMI | Journal | Unspecified | Interview and clinical examination | 60+ | Male and female | 63·7 | point prevalence |
| Chopra, 2001 | India | South Asia | LMI | Journal | All joints | Clinical | 15+ | Male and female |  | point prevalence |
| Courage, 2017 | Nigeria | SSA | LMI | Journal | All joints | Clinical - ACR criteria | 15+ | Male and female |  | point prevalence |
| Dans, 1997 | Philippines | East Asia and Pacific | LMI | Conference abstract | All joints | Clinical - ACR criteria | adults' | Male and female |  | point prevalence |
| Divengi Nzambi, 2013 | DR Congo | SSA | Low income | Conference abstract | All joints | not stated | not stated | Male and female |  | point prevalence |
| Farooqi, 1998 | Pakistan | South Asia | LMI | Journal | Knees | COPCORD methodology | 15+ | Male and female |  | point prevalence |
| Gauri, 2010 | India | South Asia | LMI | Conference abstract | All joints | COPCORD methodology | not stated | Male and female |  | point prevalence |
| Haq, 2005 (rural) | Bangladesh | South Asia | LMI | Journal | Knees | COPCORD methodology | 15+ | Male and female | 49·96 | point prevalence |
| Haq, 2005 (Urban) | Bangladesh | South Asia | LMI | Journal | Knees | COPCORD methodology | 15+ | Male and female | 49·96 | point prevalence |
| Haq, 2005 (Slum) | Bangladesh | South Asia | LMI | Journal | Knees | COPCORD methodology | 15+ | Male and female | 49·96 | point prevalence |
| Haq, 2008 | Bangladesh | South Asia | LMI | Journal | Knees | ACR criteria | 15+ | Male and female | 50·7 | incidence |
| Hien, 2014 | Burkina Faso | SSA | Low income | Journal | All joints | medical records, hstory and exam | 60+ | Male and female | 44·7 | point prevalence |
| Ho-Pham, 2014 | Vietnam | East Asia and Pacific | LMI | Journal | Knees | ACR criteria with Kellgren-Lawrence grading for XRs | 40+ | Male and female | 74·2 | point prevalence |
| Ho-Pham, 2015 | Vietnam | East Asia and Pacific | LMI | Journal | Spine | Questionnaire (?validated) and XR - Kellgren-Lawrence criteria | 40+ | Male and female | 74·2 | point prevalence |
| Hoa, 2003 | Vietnam | East Asia and Pacific | LMI | Journal | All joints | COPCORD methodology | 16+ | Male and female | 53·7 | point prevalence |
| Jamshidi, 2018 | India and Iran | South Asia and Middle East and North Africa | LMI and UMI | Conference abstract | All joints | COPCORD methodology | 16+ | Male and female |  | point prevalence |
| Kalichman, 2011 | USSR | Europe and Central Asia |  | Journal | Hands | XRs using Kellgren Lawrence (KL) criteria | 18+ | Male and female |  | point prevalence |
| Kumar, 2018 | India | South Asia | LMI | Journal | All joints | questionnaire, exam and XR (COPCORD methodology) | 0+ | Male and female |  | point prevalence |
| Mehrotra, 2010 | India | South Asia | LMI | Conference abstract | Knees | Questionnaire self report, a few were examined but not all (ACR criteria) | 0+ | male and female |  | point prevalence |
| Namali, 2011 | Sri Lanka | South Asia | LMI | Conference abstract | Knees | Questionnaire ACR criteria | 50+ | Male and female |  | point prevalence |
| Ogunniyi, 2001 | Nigeria | SSA | LMI | Journal | All joints | Questionnaire, exam, and XR | 65+ | Male and female | 64·9 | point prevalence |
| Pal, 2016 | India | South Asia | LMI | Journal | Knees | Questionnaire, and XR (KL criteria) | 40+ | Male and female |  | point prevalence |
| Patel, 2011 | India | South Asia | LMI | Journal | All joints | Questionnaire | not reported | Male and female |  | not clear |
| Paul, 2013 | India | South Asia | LMI | Journal | All joints | COPCORD - Questionnaire, exam (ACR criteria) and XR (KL criteria) | 15+ | Male and female | 50·7 | point prevalence |
| PrashansanieHettihewa, 2018 | Sri Lanka | South Asia | LMI | Journal | Knees | clinical - ACR criteria followed by XR (KL criteria) | 50+ | Female | 100 | point prevalence |
| Rao, 2018 (Rural) | India | South Asia | LMI | Journal | Knees | History only | women - 40+; men - 50+ | Male and female | 58·1 | not clear |
| Rao, 2018 (Semi-urban) | India | South Asia | LMI | Journal | Knees | History only | women - 40+; men - 50+ | Male and female | 59·8 | not clear |
| Salve, 2010 | India | South Asia | LMI | Journal | Knees | Clinical exam - ACR criteria | 40+ | Female | 100 | point prevalence |
| Smythe, 2017 | Cameroon | SSA | LMI | Journal | All joints | questionnaire followed by exam | all ages | Male and female | 59 | point prevalence |
| Venkatachalam, 2018 | India | South Asia | LMI | Journal | Knees | ACR criteria | 18+ | Male and female | 63·4 | point prevalence |
| Wigley, 1991 | Philippines | East Asia and Pacific | LMI | Journal | Knees | Questionnaire, examination | 15+ | Male and female | 49·5 | point prevalence |

**Supplementary table S3: Risk of bias table**

| **Study** | **Country** | **Was target population a close representation** | **Was sampling frame a true representation** | **Was random selection used?** | **Was the likelihood of nonresponse bias minimal** | **Were data directly from subjects?** | **Was the case definition acceptable?** | **Was the method shown to have validity and reliability?** | **Was same mode of data collection used** | **Was the length of the shortest prevalence period for the parameter of interest appropriate?** | **Were the numerator(s) and denominator(s) appropriate and clearly reported?** | **Risk of bias score** |  |
| --- | --- | --- | --- | --- | --- | --- | --- | --- | --- | --- | --- | --- | --- |
| Abegunde, 2013 | Nigeria | 0 | 1 | 1 | 1 | 0 | 1 | 0 | 1 | 1 | 1 | 7 |  |
| Adekanla, 2007 | Nigeria | 0 | 1 | 1 | 0 | 1 | 1 | 1 | 1 | 1 | 1 | 8 |  |
| Akinpelu, 2009 | Nigeria | 0 | 1 | 1 | 0 | 1 | 1 | 1 | 1 | 1 | 1 | 8 |  |
| Alonge, 2009 | Nigeria | 0 | 1 | 0 | 0 | 1 | 1 | 1 | 1 | 1 | 1 | 7 |  |
| Ananto, 2018 | Indonesia | 0 | 1 | 1 | 0 | 1 | 1 | 1 | 1 | 1 | 1 | 8 |  |
| Ara, 2014 | Bangladesh | 0 | 0 | 0 | 1 | 1 | 1 | 1 | 1 | 1 | 1 | 7 |  |
| Bella, 1993 | Nigeria | 1 | 1 | 0 | 0 | 1 | 0 | 0 | 1 | 1 | 1 | 6 |  |
| Chopra, 2001 | India | 0 | 1 | 0 | 1 | 1 | 1 | 1 | 1 | 1 | 1 | 8 |  |
| Courage, 2017 | Nigeria | 0 | 1 | 1 | 0 | 1 | 1 | 1 | 1 | 1 | 0 | 7 |  |
| Dans, 1997 | Philippines | 0 | 1 | 1 | 1 | 1 | 1 | 1 | 1 | 1 | 1 | 9 |  |
| Divengi Nzambi, 2013 | Democratic Republic of Congo (DRC) | 0 | 0 | 0 | 0 | 1 | 0 | 0 | 1 | 0 | 1 | 3 |  |
| Farooqi, 1998 | Pakistan | 1 | 1 | 1 | 1 | 1 | 1 | 1 | 1 | 1 | 1 | 10 |  |
| Gauri, 2010 | India | 0 | 0 | 0 | 0 | 1 | 1 | 1 | 1 | 1 | 1 | 6 |  |
| Haq, 2005 | Bangladesh | 1 | 1 | 1 | 0 | 1 | 1 | 1 | 1 | 1 | 1 | 9 |  |
| Haq, 2008 | Bangladesh | 0 | 1 | 1 | 0 | 1 | 1 | 1 | 1 | 1 | 1 | 8 |  |
| Hien, 2014 | Burkina Faso | 0 | 1 | 1 | 0 | 1 | 0 | 0 | 1 | 1 | 1 | 6 |  |
| Ho-Pham, 2014 | Vietnam | 0 | 0 | 1 | 1 | 1 | 1 | 1 | 1 | 1 | 1 | 8 |  |
| Ho-Pham, 2015 | Vietnam | 0 | 0 | 1 | 1 | 1 | 1 | 1 | 1 | 1 | 1 | 8 |  |
| Hoa, 2003 | Vietnam | 0 | 1 | 1 | 1 | 1 | 1 | 1 | 1 | 1 | 1 | 9 |  |
| **Jamshidi, 2018** | **India** | **0** | **1** | **0** | **0** | **1** | **1** | **1** | **1** | **1** | **1** | **7** |  |
| Kalichman, 2011 | Ukraine | 0 | 0 | 0 | 0 | 1 | 1 | 1 | 1 | 1 | 1 | 6 |  |
| Kumar, 2018 | India | 0 | 1 | 1 | 1 | 1 | 1 | 1 | 1 | 1 | 1 | 9 |  |
| **Mehrotra, 2010** | **India** | **0** | **0** | **0** | **0** | **1** | **1** | **0** | **1** | **1** | **0** | **4** |  |
| Namali, 2011 | Sri Lanka | 0 | 1 | 1 | 1 | 1 | 1 | 1 | 1 | 1 | 0 | 8 |  |
| Ogunniyi, 2001 | Nigeria | 0 | 1 | 0 | 0 | 1 | 1 | 0 | 1 | 1 | 1 | 6 |  |
| Pal, 2016 | India | 1 | 1 | 1 | 0 | 1 | 1 | 1 | 1 | 1 | 0 | 8 |  |
| Patel, 2011 | India | 0 | 0 | 0 | 0 | 1 | 0 | 0 | 1 | 0 | 0 | 2 |  |
| Paul, 2013 | India | 0 | 1 | 1 | 1 | 1 | 1 | 1 | 1 | 1 | 0 | 8 |  |
| Prashansanie Hettihewa, 2018 | Sri Lanka | 0 | 1 | 1 | 1 | 1 | 1 | 1 | 1 | 1 | 1 | 9 |  |
| Rao, 2018 | India | 0 | 0 | 0 | 0 | 1 | 0 | 1 | 1 | 1 | 0 | 4 |  |
| Salve, 2010 | India | 0 | 1 | 1 | 0 | 1 | 1 | 1 | 1 | 1 | 1 | 8 |  |
| Smythe, 2017 | Cameroon | 0 | 1 | 1 | 1 | 1 | 0 | 0 | 1 | 1 | 1 | 7 |  |
| Venkatachalam, 2018 | India | 0 | 1 | 1 | 1 | 1 | 1 | 1 | 1 | 1 | 1 | 9 |  |
| Wigley, 1991 | Philippines | 0 | 1 | 0 | 0 | 1 | 0 | 0 | 1 | 1 | 1 | 5 |  |
