# Supplementary material for: Prevalence of osteoarthritis in lower middle- and low-income countries: a systematic review and meta-analysis
Source: Rheumatol Int. 2021 Apr 27;41(7):1221–31. doi: 10.1007/s00296-021-04838-y (PMC8164595; doi:10.1007/s00296-021-04838-y)

**Supplementary figure S1: Secular trends in OA prevalence estimates**


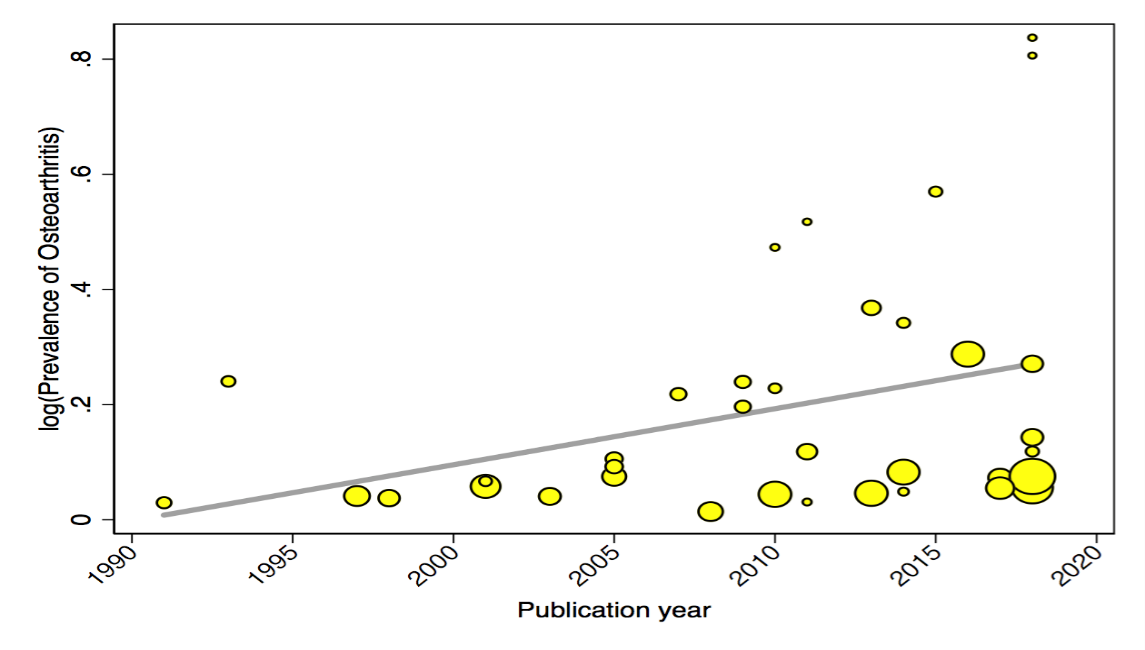


**Supplementary figure S2: Association between sample size and prevalence estimates**


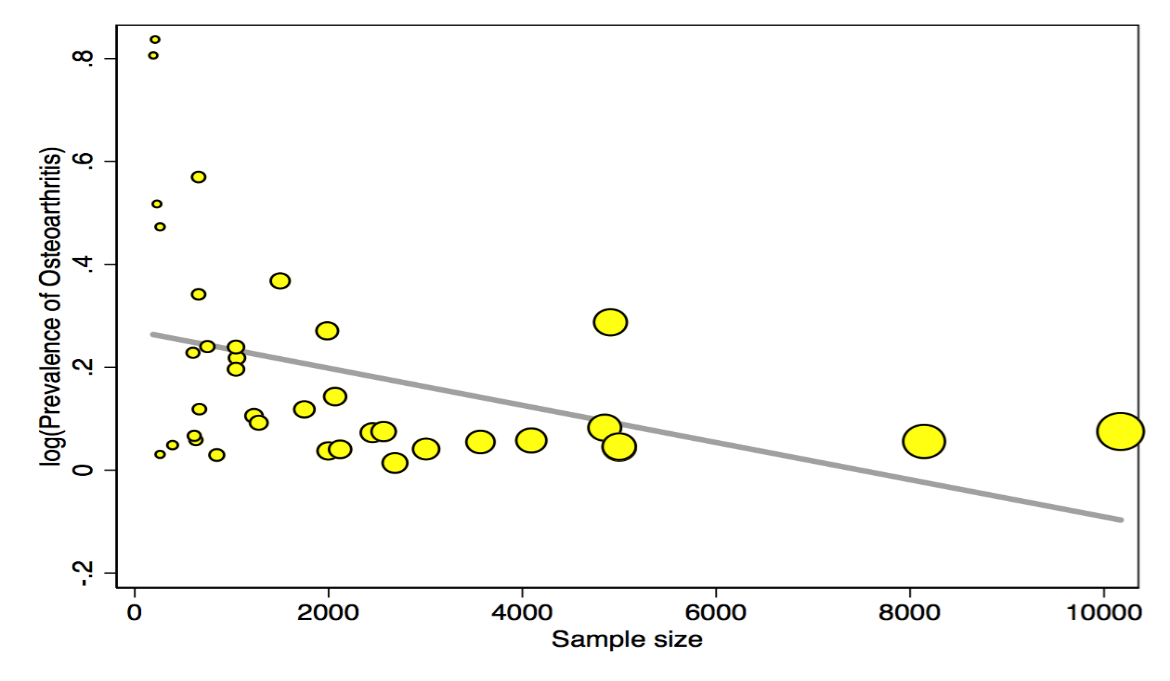


**Supplementary figure S3: Association between quality and prevalence estimates**


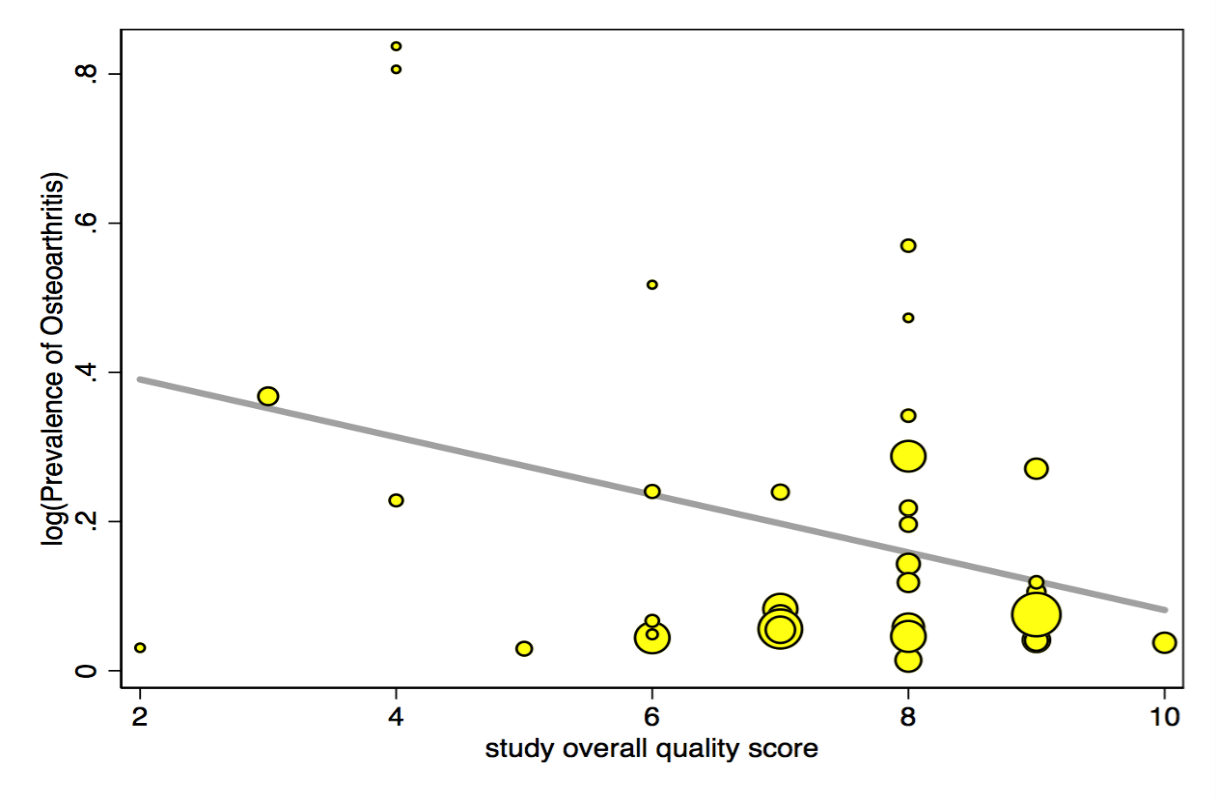


**Supplementary figure S4: Association between percentage female and prevalence estimates**


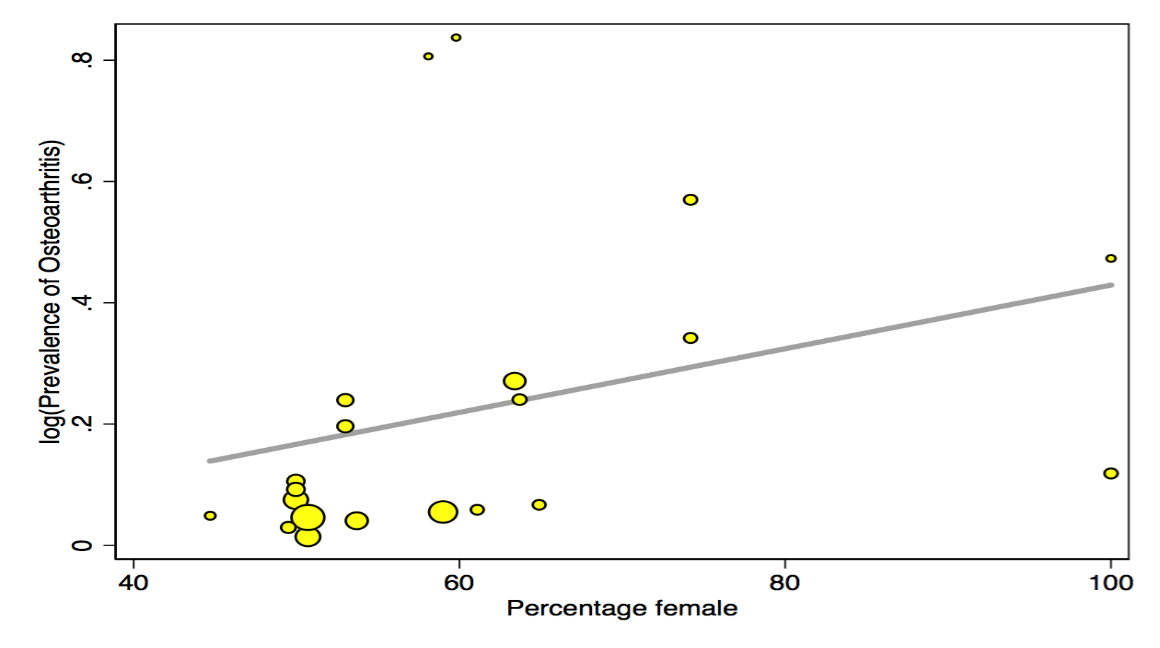

Supplement: Supplementary file 2 — Supplementary file2 Supplementary table S2 SSA Sub-Saharan Africa, LMI Lower and middle-income countries, ACR American College of Rheumatology, KL Kellgren Lawrence , XR X-ray (DOCX 26 KB) [file 296_2021_4838_MOESM1_ESM.docx]
