# Supplementary material for: Prevalence of osteoarthritis in lower middle- and low-income countries: a systematic review and meta-analysis
Source: Rheumatol Int. 2021 Apr 27;41(7):1221–31. doi: 10.1007/s00296-021-04838-y (PMC8164595; doi:10.1007/s00296-021-04838-y)
Supplement: Supplementary file 3 — Supplementary file3 (DOCX 40 KB) [file 296_2021_4838_MOESM3_ESM.docx]

**PREVALENCE OF OSTEOARTHRITIS IN LOW AND MIDDLE INCOME COUNTRIES**

Search results summary table

| Database | Date | Hits |
| --- | --- | --- |
| Web of Science | 6/11/18 | 2526 |
| CINAHL Plus | 6/11/18 | 889 |
| Medline (Ovid) | 6/11/18 | 2000 |
| Embase (Ovid) | 6/11/18 | 1999 |
|  |  | 7414 |

Web of science 06/11/2018

# 33 2,526 #32 AND #23 AND #11

# 32 4,293,126 #31 OR #30 OR #29 OR #28 OR #27 OR #26 OR #25 OR #24

# 31 149,240 ts=("developing countr*" OR "developing econom*" OR "developing nation" OR "developing nations" OR "middle income countr*" OR "middle income econom*" OR "middle income nation" OR "middle income nations" OR "underdeveloped countr*" OR "underdeveloped economy*" OR "underdeveloped nation" OR "underdeveloped nations" OR "under developed countr*" OR "under developed econom*" OR "under developed nation" OR "under developed nations" OR "3rd world countr*" OR "3rd world econom*" OR "3rd world nation" OR "3rd world nations" OR "third world countr*" OR "third world econom*" OR "third world nation" OR "third world nations" OR "LAMI countr*" OR “LAMI nation” OR “LAMI nations” OR “LAMI econom*” OR "LMIC countr*" OR “LMIC nation” OR “LMIC nations” OR “LMIC econom*” OR "poor income countr*" OR “poor income nation” OR “poor income nations” OR “poor income economy*” OR "low* income countr*" OR “low income nation” OR “low income nations” OR “low income econom*” OR "less* developed countr*" OR “less* developed nation” OR “less developed nations” OR “less developed econom*” OR "least developed countr*" OR “least developed nation” OR “least developed nations” OR “least developed econom*”)

# 30 3,699,954 ts=(Afghan* or Angola* or Armenia* or Bangladesh* or Benin* or Bhutan* or Bolivia* or “Burkina Faso*” or “Upper Volta*” or Burundi* or “Cabo Verd*” or “Cape Verd*” or Cambodia* or Cameroon* or “Central African Republic*” or Chad* or Comoros or Comorian or Congo* or “Cote d'Ivoire” or “Ivory Coast” or Ivorian* or Djibouti* or Egypt* or “El Salvador*” or Eritrea* or Ethiopia* or Gambia* or Georgia* or Ghana* or Guatemala* or Guinea* or Haiti* or Hondura* or India* or Indonesia* or Jordan* or Kenya* or Kiribati* or Korea* or Lesotho or Mosotho or Basotho or Liberia* or Madagasca* or Malagasy or Kosov* or Kyrgyz* or Lao* or Malawi* or Mali or Mali's or Malian* or Mauritania* or Micronesia* or Moldov* or Mongolia* or Morocc* or Mozambi* or Myanmar* or Burma* or Burmese* or Nepal* or Nicaragua* or Niger* or Pakistan* or PNG* or Philippines* or Filipino* or Rwanda* or "Sao Tome*" or Principe* or Senegal* or “Sierra Leone”* or “Solomon Island*” or Somalia* or Sudan* or "Sri Lanka*" or Swaziland* or Syria* or Tajik* or Tanzania* or Timor* or Togo* or Tunisia* or Uganda* or Ukrain* or Uzbek* or Vanuatu* or Vietnam* or “West Bank*” or Gaza* or Palestin* or Yemen* or Zambia* or Zimbabwe* or Albania* or Algeria* or Argentin* or Samoa* or Azerbaijan* or Belarus* or Belize* or Bosnia* or Herzegovia* or Botswana* or Brazil* or Bulgaria* or China* or Chinese* or Colombia* or “Costa Rica*” or Croatia* or Cuba* or Dominica* or Ecuador* or Fiji* or Gabon* or Grenad* or Guyan* or Iran* or Iraq* or Jamaica* or Kazakh* or Leban* or Libya* or Macedonia* or Malaysia* or Maldiv* or “Marshall Island”* or Mauriti* or Mexic* or Montenegr* or Namibia* or Nauru* or Panama* or Paraguay* or Peru* or Romania* or Russia* or Serbia* or “South Africa*” or Suriname* or Lucia* or Vincent* or Thai* or Tonga* or Turk* or Turkmen* or Tuvalu* or Venezuela*)

# 29 36,796 ts="East* Europe*"

# 28 43,806 ts=(Caribbean* or "West Indies")

# 27 137,677 ts=((Latin or Central or South) near/5 America*)

# 26 50,821 ts=((Middle or Far) near/5 East*)

# 25 524,779 ts=("africa*")

# 24 300,305 ts=("asia*")

# 23 4,696,878 #22 OR #21 OR #20 OR #19 OR #18 OR #17 OR #16 OR #15 OR #14 OR #13 OR #12

# 22 462,654 ts=epidemiol*

# 21 774,534 ts=trend*

# 20 1,084,778 ts=long-term

# 19 1,295,742 ts=progress*

# 18 13,157 ts=(disease* near/3 pattern*)

# 17 247,421 ts=determinants*

# 16 7,096 ts=(disease* near/3 frequenc*)

# 15 32,339 ts=(disease* near/3 rate*)

# 14 4,502 ts=occurenc*

# 13 640,401 ts=incidenc*

# 12 855,710 ts=preval*

# 11 129,230 #10 OR #9 OR #8 OR #7 OR #6 OR #5 OR #4 OR #3 OR #2 OR #1

# 10 221 ts=(thumb* near/3 (pain or painful)).

# 9 607 ts=(finger* near/3 (pain or painful)).

# 8 11,256 ts=(joint* near/3 (pain or painful)).

# 7 2,634 ts=(hand* near/3 (pain or painful)).

# 6 5,265 ts=(hip near/3 (pain or painful)).

# 5 10,868 ts=(knee near/3 (pain or painful)).

# 4 4,832 ts=(degenerative near/3 (arthritis or joint or joints)).

# 3 3,453 ts=arthrosis

# 2 36,781 ts=oa

# 1 85,801 ts=osteoarthr*

CINAHLPlus (EBSCO) 06/11/18

|  |  |  |  |
| --- | --- | --- | --- |
| S100 | S12 AND S98 AND S99 | (889) |  |
|  |  |  |  |
|  |  |  |  |
| S99 | S27 OR S28 OR S29 OR S30 OR S31 OR S32 OR S33 OR S34 OR S35 OR S36 OR S37 OR S38 OR S39 OR S40 OR S41 OR S42 OR S43 OR S44 OR S45 OR S46 OR S47 OR S48 OR S49 OR S50 OR S51 OR S52 OR S53 OR S54 OR S55 OR S56 OR S57 OR S58 OR S59 OR S60 OR S61 OR S62 OR S63 OR S64 OR S65 OR S66 OR S67 OR S68 OR S69 OR S70 OR S71 OR S72 OR S73 OR S74 OR S75 OR S76 OR S77 OR S78 OR S79 OR S80 OR S81 OR S82 OR S83 OR S84 OR S85 OR S86 OR S87 OR S88 OR S89 OR S90 OR S91 OR S92 OR S93 OR S94 OR S95 OR S96 OR S97 | (533,269) |  |
|  |  |  |  |
|  |  |  |  |
| S98 | S13 OR S14 OR S15 OR S16 OR S17 OR S18 OR S19 OR S20 OR S21 OR S22 OR S23 OR S24 OR S25 OR S26 | (824,685) |  |
|  |  |  |  |
|  |  |  |  |
| S97 | TI ( Tuvalu* or Venezuela* ) OR AB ( Tuvalu* or Venezuela* ) | (565) |  |
|  |  |  |  |
|  |  |  |  |
| S96 | TI ( Tonga* or Turk* or Turkmen* ) OR AB ( Tonga* or Turk* or Turkmen* ) | (11,313) |  |
|  |  |  |  |
|  |  |  |  |
| S95 | TI ( Lucia* or Vincent* or Thai* ) OR AB ( Lucia* or Vincent* or Thai* ) | (7,058) |  |
|  |  |  |  |
|  |  |  |  |
| S94 | TI ( Serbia* or "South Africa*" or Suriname* ) OR AB ( Serbia* or "South Africa*" or Suriname* ) | (12,742) |  |
|  |  |  |  |
|  |  |  |  |
| S93 | TI ( Peru* or Romania* or Russia* ) OR AB ( Peru* or Romania* or Russia* ) | (7,132) |  |
|  |  |  |  |
|  |  |  |  |
| S92 | TI ( Nauru* or Panama* or Paraguay* ) OR AB ( Nauru* or Panama* or Paraguay* ) | (576) |  |
|  |  |  |  |
|  |  |  |  |
| S91 | TI ( Mexic* or Montenegr* or Namibia* ) OR AB ( Mexic* or Montenegr* or Namibia* ) | (13,098) |  |
|  |  |  |  |
|  |  |  |  |
| S90 | TI ( Maldiv* or "Marshall Island*" or Mauriti* ) OR AB ( Maldiv* or "Marshall Island*" or Mauriti* ) | (348) |  |
|  |  |  |  |
|  |  |  |  |
| S89 | TI ( Libya* or Macedonia* or Malaysia* ) OR AB ( Libya* or Macedonia* or Malaysia* ) | (4,375) |  |
|  |  |  |  |
|  |  |  |  |
| S88 | TI ( Jamaica* or Kazakh* or Leban* ) OR AB ( Jamaica* or Kazakh* or Leban* ) | (2,494) |  |
|  |  |  |  |
|  |  |  |  |
| S87 | TI ( Guyan* or Iran* or Iraq ) OR AB ( Guyan* or Iran* or Iraq ) | (15,769) |  |
|  |  |  |  |
|  |  |  |  |
| S86 | TI ( Fiji* or Gabon* or Grenad* ) OR AB ( Fiji* or Gabon* or Grenad* ) | (683) |  |
|  |  |  |  |
|  |  |  |  |
| S85 | TI ( Cuba* or Dominica* or Ecuador* ) OR AB ( Cuba* or Dominica* or Ecuador* ) | (2,964) |  |
|  |  |  |  |
|  |  |  |  |
| S84 | TI ( Colombia* or "Costa Rica*" or Croatia* ) OR AB ( Colombia* or "Costa Rica*" or Croatia* ) | (4,178) |  |
|  |  |  |  |
|  |  |  |  |
| S83 | TI ( Brazil* or Bulgaria* or China* or Chinese* ) OR AB ( Brazil* or Bulgaria* or China* or Chinese* ) | (75,680) |  |
|  |  |  |  |
|  |  |  |  |
| S82 | TI ( Belize* or Bosnia* or Herzegovia* or Botswana* ) OR AB ( Belize* or Bosnia* or Herzegovia* or Botswana* ) | (1,323) |  |
|  |  |  |  |
|  |  |  |  |
| S81 | TI ( Samoa* or Azerbaijan* or Belarus* ) OR AB ( Samoa* or Azerbaijan* or Belarus* ) | (749) |  |
|  |  |  |  |
|  |  |  |  |
| S80 | TI ( Albania* or Algeria* or Argentin* ) OR AB ( Albania* or Algeria* or Argentin* ) | (2,442) |  |
|  |  |  |  |
|  |  |  |  |
| S79 | TI ( Yemen* or Zambia* or Zimbabwe* ) OR AB ( Yemen* or Zambia* or Zimbabwe* ) | (3,167) |  |
|  |  |  |  |
|  |  |  |  |
| S78 | TI ( "West Bank*" or Gaza* or Palestin* ) OR AB ( "West Bank*" or Gaza* or Palestin* ) | (1,341) |  |
|  |  |  |  |
|  |  |  |  |
| S77 | TI ( Uzbek* or Vanuatu* or Vietnam* ) OR AB ( Uzbek* or Vanuatu* or Vietnam* ) | (4,553) |  |
|  |  |  |  |
|  |  |  |  |
| S76 | TI ( Tunisia* or Uganda* or Ukrain* ) OR AB ( Tunisia* or Uganda* or Ukrain* ) | (5,670) |  |
|  |  |  |  |
|  |  |  |  |
| S75 | TI ( Tanzania* or Timor* or Togo* ) OR AB ( Tanzania* or Timor* or Togo* ) | (3,414) |  |
|  |  |  |  |
|  |  |  |  |
| S74 | TI ( Swaziland* or Syria* or Tajik* ) OR AB ( Swaziland* or Syria* or Tajik* ) | (1,527) |  |
|  |  |  |  |
|  |  |  |  |
| S73 | TI ( Somalia* or Sudan* or "Sri Lanka*" ) OR AB ( Somalia* or Sudan* or "Sri Lanka*" ) | (3,254) |  |
|  |  |  |  |
|  |  |  |  |
| S72 | TI ( Senegal* or "Sierra Leone*" or "Solomon Island*" ) OR AB ( Senegal* or "Sierra Leone*" or "Solomon Island*" ) | (1,444) |  |
|  |  |  |  |
|  |  |  |  |
| S71 | TI ( Rwanda* or "Sao Tome*" or Principe* ) OR AB ( Rwanda* or "Sao Tome*" or Principe* ) | (1,074) |  |
|  |  |  |  |
|  |  |  |  |
| S70 | TI ( Pakistan* or PNG* or Philippines* or Filipino* ) OR AB ( Pakistan* or PNG* or Philippines* or Filipino* ) | (6,619) |  |
|  |  |  |  |
|  |  |  |  |
| S69 | TI ( Nepal* or Nicaragua* or Niger* ) OR AB ( Nepal* or Nicaragua* or Niger* ) | (9,544) |  |
|  |  |  |  |
|  |  |  |  |
| S68 | TI ( Morocc* or Mozambi* or Myanmar* or Burma* or Burmese* ) OR AB ( Morocc* or Mozambi* or Myanmar* or Burma* or Burmese* ) | (2,637) |  |
|  |  |  |  |
|  |  |  |  |
| S67 | TI ( Micronesia* or Moldov* or Mongolia* ) OR AB ( Micronesia* or Moldov* or Mongolia* ) | (929) |  |
|  |  |  |  |
|  |  |  |  |
| S66 | TI ( Malawi* or Mali* or Mauritania* ) OR AB ( Malawi* or Mali* or Mauritania* ) | (57,355) |  |
|  |  |  |  |
|  |  |  |  |
| S65 | TI ( Kosov* or Kyrgyz* or Lao* ) OR AB ( Kosov* or Kyrgyz* or Lao* ) | (1,061) |  |
|  |  |  |  |
|  |  |  |  |
| S64 | TI ( Liberia* or Madagasca* or Malagasy ) OR AB ( Liberia* or Madagasca* or Malagasy ) | (847) |  |
|  |  |  |  |
|  |  |  |  |
| S63 | TI ( Lesotho or Mosotho or Basotho ) OR AB ( Lesotho or Mosotho or Basotho ) | (210) |  |
|  |  |  |  |
|  |  |  |  |
| S62 | TI ( Kenya* or Kiribati* or Korea* ) OR AB ( Kenya* or Kiribati* or Korea* ) | (20,355) |  |
|  |  |  |  |
|  |  |  |  |
| S61 | TI ( India* or Indonesia* or Jordan* ) OR AB ( India* or Indonesia* or Jordan* ) | (37,956) |  |
|  |  |  |  |
|  |  |  |  |
| S60 | TI ( Guinea* or Haiti* or Hondura* ) OR AB ( Guinea* or Haiti* or Hondura* ) | (4,924) |  |
|  |  |  |  |
|  |  |  |  |
| S59 | TI ( Georgia* or Ghana* or Guatemala* ) OR AB ( Georgia* or Ghana* or Guatemala* ) | (7,194) |  |
|  |  |  |  |
|  |  |  |  |
| S58 | TI ( Eritrea* or Ethiopia* or Gambia* ) OR AB ( Eritrea* or Ethiopia* or Gambia* ) | (4,003) |  |
|  |  |  |  |
|  |  |  |  |
| S57 | TI ( Djibouti* or Egypt* or "El Salvador*" ) OR AB ( Djibouti* or Egypt* or "El Salvador*" ) | (3,147) |  |
|  |  |  |  |
|  |  |  |  |
| S56 | TI ( "Cote d'Ivoire" or "Ivory Coast" or Ivorian* ) OR AB ( "Cote d'Ivoire" or "Ivory Coast" or Ivorian* ) | (136) |  |
|  |  |  |  |
|  |  |  |  |
| S55 | TI ( Chad* or Comoros or Comorian or Congo* ) OR AB ( Chad* or Comoros or Comorian or Congo* ) | (2,269) |  |
|  |  |  |  |
|  |  |  |  |
| S54 | TI ( Cambodia* or Cameroon* or "Central African Republic*" ) OR AB ( Cambodia* or Cameroon* or "Central African Republic*" ) | (2,538) |  |
|  |  |  |  |
|  |  |  |  |
| S53 | TI ( Burundi* or "Cabo Verd*" or "Cape Verd*" ) OR AB ( Burundi* or "Cabo Verd*" or "Cape Verd*" ) | (207) |  |
|  |  |  |  |
|  |  |  |  |
| S52 | TI ( Bolivia* or "Burkina Faso*" or "Upper Volta*" ) OR AB ( Bolivia* or "Burkina Faso*" or "Upper Volta*" ) | (1,330) |  |
|  |  |  |  |
|  |  |  |  |
| S51 | TI ( Bangladesh* or Benin* or Bhutan* ) OR AB ( Bangladesh* or Benin* or Bhutan* ) | (4,402) |  |
|  |  |  |  |
|  |  |  |  |
| S50 | TI ( Afghan* or Angola* or Armenia* ) OR AB ( Afghan* or Angola* or Armenia* ) | (3,341) |  |
|  |  |  |  |
|  |  |  |  |
| S49 | TI "east* europe*" OR AB "east* europe*" | (1,647) |  |
|  |  |  |  |
|  |  |  |  |
| S48 | TI africa* OR AB africa* | (56,874) |  |
|  |  |  |  |
|  |  |  |  |
| S47 | TI ( (middle or far) W0 east* ) OR AB ( (middle or far) W0 east* ) | (3,097) |  |
|  |  |  |  |
|  |  |  |  |
| S46 | TI "west* asia*" OR AB "west* asia*" | (60) |  |
|  |  |  |  |
|  |  |  |  |
| S45 | TI ( ("south east") W0 asia* ) OR AB ( ("south east") W0 asia* ) | (778) |  |
|  |  |  |  |
|  |  |  |  |
| S44 | TI south* W0 asia* OR AB south* W0 asia* | (4,473) |  |
|  |  |  |  |
|  |  |  |  |
| S43 | (MH "Europe, Eastern+") | (20,117) |  |
|  |  |  |  |
|  |  |  |  |
| S42 | TI ( (central or latin or south) W0 america* ) OR AB ( (central or latin or south) W0 america* ) | (6,495) |  |
|  |  |  |  |
|  |  |  |  |
| S41 | TI ( carribean* or "west indies") OR AB ( carribean* or "west indies") | (227) |  |
|  |  |  |  |
|  |  |  |  |
| S40 | (MH "Africa+") | (66,315) |  |
|  |  |  |  |
|  |  |  |  |
| S39 | (MH "Asia+") | (222,236) |  |
|  |  |  |  |
|  |  |  |  |
| S38 | TI ( ("least developed" W0 (countr* or nation* or econom*)) ) OR AB ( ("least developed" W0 (countr* or nation* or econom*)) ) | (57) |  |
|  |  |  |  |
|  |  |  |  |
| S37 | TI ( ("less* developed" W0 (countr* or nation* or econom*)) ) OR AB ( ("less* developed" W0 (countr* or nation* or econom*)) | (247) |  |
|  |  |  |  |
|  |  |  |  |
| S36 | TI ( ("low* income" W0 (countr* or nation* or econom*)) ) OR AB ( ("low* income" W0 (countr* or nation* or econom*)) ) | (2,158) |  |
|  |  |  |  |
|  |  |  |  |
| S35 | TI ( ("poor income" W0 (countr* or nation* or econom*)) ) OR AB ( ("poor income" W0 (countr* or nation* or econom*)) ) | (1) |  |
|  |  |  |  |
|  |  |  |  |
| S34 | TI ( (LMIC W0 (countr* or nation* or econom*)) ) OR AB ( (LMIC W0 (countr* or nation* or econom*)) ) | (17) |  |
|  |  |  |  |
|  |  |  |  |
| S33 | TI ( (LAMI W0 (countr* or nation* or econom*)). ) OR AB ( (LAMI W0 (countr* or nation* or econom*)). ) | (11) |  |
|  |  |  |  |
|  |  |  |  |
| S32 | TI ( ( ("third world" W0 (countr* or nation* or econom*)) ) OR AB ( ( ("third world" W0 (countr* or nation* or econom*)) ) | (153) |  |
|  |  |  |  |
|  |  |  |  |
| S31 | TI ( ("3rd world" W0 (countr* or nation* or economy*)) ) OR AB ( ("3rd world" W0 (countr* or nation* or economy*)) ) | (1) |  |
|  |  |  |  |
|  |  |  |  |
| S30 | TI ( (("under developed" or underdeveloped) W0 (countr* or nation* or economy*)) ) OR AB ( (("under developed" or underdeveloped) W0 (countr* or nation* or econom*)).ti,ab. ) | (15) |  |
|  |  |  |  |
|  |  |  |  |
| S29 | TI ((developing W0 (countr* or nation* or econom*)) OR AB ((developing W0 (countr* or nation* or econom*)) | (12,116) |  |
|  |  |  |  |
|  |  |  |  |
| S28 | TI ( ("middle income*" W0 (countr* or nation* or econom*)) ) OR AB ( ("middle income*" W0 (countr* or nation* or econom*)) ) | (5,619) |  |
|  |  |  |  |
|  |  |  |  |
| S27 | (MH "Developing Countries") | (16,374) |  |
|  |  |  |  |
|  |  |  |  |
| S26 | TI epidemiol* OR AB epidemiol* | (63,042) |  |
|  |  |  |  |
|  |  |  |  |
| S25 | (MM "Epidemiology+") | (121,705) |  |
|  |  |  |  |
|  |  |  |  |
| S24 | TI trend* OR AB trend* | (84,328) |  |
|  |  |  |  |
|  |  |  |  |
| S23 | TI long-term OR AB long-term | (137,957) |  |
|  |  |  |  |
|  |  |  |  |
| S22 | TI progress* OR AB progress* | (140,697) |  |
|  |  |  |  |
|  |  |  |  |
| S21 | TI disease* n3 pattern** OR AB disease* n3 pattern* | (1,956) |  |
|  |  |  |  |
|  |  |  |  |
| S20 | TI determinant* OR AB determinant* | (35,746) |  |
|  |  |  |  |
|  |  |  |  |
| S19 | TI disease n3 frequenc* OR AB disease n3 frequenc* | (1,069) |  |
|  |  |  |  |
|  |  |  |  |
| S18 | TI disease n3 rate* OR AB disease n3 rate* | (7,565) |  |
|  |  |  |  |
|  |  |  |  |
| S17 | TI occurrence* OR AB occurrence* | (41,104) |  |
|  |  |  |  |
|  |  |  |  |
| S16 | TI inciden* OR AB inciden* | (151,492) |  |
|  |  |  |  |
|  |  |  |  |
| S15 | (MH "Incidence") | (55,762) |  |
|  |  |  |  |
|  |  |  |  |
| S14 | TI preval* OR AB preval* | (161,874) |  |
|  |  |  |  |
|  |  |  |  |
| S13 | (MH "Prevalence") | (74,457) |  |
|  |  |  |  |
|  |  |  |  |
| S12 | S1 OR S2 OR S3 OR S4 OR S5 OR S6 OR S7 OR S8 OR S9 OR S10 OR S11 | (42,915) |  |
|  |  |  |  |
|  |  |  |  |
| S11 | TI thumb* n3 pain* OR AB thumb* n3 pain* | (101) |  |
|  |  |  |  |
|  |  |  |  |
| S10 | TI finger* n3 pain* OR AB finger* n3 pain* | (230) |  |
|  |  |  |  |
|  |  |  |  |
| S9 | TI joint* n3 pain* OR AB joint* n3 pain* | (4,370) |  |
|  |  |  |  |
|  |  |  |  |
| S8 | TI hand* n3 pain* OR AB hand* n3 pain* | (1,214) |  |
|  |  |  |  |
|  |  |  |  |
| S7 | TI hip n3 pain* OR AB hip n3 pain* | (2,794) |  |
|  |  |  |  |
|  |  |  |  |
| S6 | TI knee n3 pain* OR AB knee n3 pain* | (5,558) |  |
|  |  |  |  |
|  |  |  |  |
| S5 | TI ( (degenerative n3 (arthritis or joint*) ) OR AB ( (degenerative n3 (arthritis or joint*) ) | (1,008) |  |
|  |  |  |  |
|  |  |  |  |
| S4 | TI arthrosis OR AB arthrosis | (671) |  |
|  |  |  |  |
|  |  |  |  |
| S3 | TI oa OR AB oa | (8,138) |  |
|  |  |  |  |
|  |  |  |  |
| S2 | TI osteoarth* OR AB osteoarth* | (22,787) |  |
|  |  |  |  |
|  |  |  |  |
| S1 | (MH "Osteoarthritis+") | (23,342) |  |
|  |  |  |  |
|  |  |  | |

**Ovid MEDLINE(R) and Epub Ahead of Print, In-Process & Other Non-Indexed Citations, Daily and Versions(R)**1946 to November 05, 2018

| Searches | Results | Type |
| --- | --- | --- |
|  |  |  |
| 1 | exp Osteoarthritis/ | 56800 |
|  |  |  |
| 2 | osteoarthr$.ti,ab,kw. | 63719 |
|  |  |  |
| 3 | OA.ti,ab,kw. | 29931 |
|  |  |  |
| 4 | arthrosis.ti,ab,kw. | 5158 |
|  |  |  |
| 5 | (degenerative adj (arthritis or joint or joints)).ti,ab,kw. | 3994 |
|  |  |  |
| 6 | (knee adj3 (pain or painful)).ti,ab,kw. | 9444 |
|  |  |  |
| 7 | (hip adj3 (pain or painful)).ti,ab,kw. | 5360 |
|  |  |  |
| 8 | (hand$ adj3 (pain or painful)).ti,ab,kw. | 2115 |
|  |  |  |
| 9 | (joint$ adj3 (pain or painful)).ti,ab,kw. | 11027 |
|  |  |  |
| 10 | (finger$ adj3 (pain or painful)).ti,ab,kw. | 579 |
|  |  |  |
| 11 | (thumb$ adj3 (pain or painful)).ti,ab,kw. | 232 |
|  |  |  |
| 12 | or/1-11 | 118080 |
|  |  |  |
| 13 | prevalence/ | 259200 |
|  |  |  |
| 14 | preval$.ti,ab,kw. | 663178 |
|  |  |  |
| 15 | incidence/ | 235683 |
|  |  |  |
| 16 | inciden$.ti,ab,kw. | 792384 |
|  |  |  |
| 17 | occurence$.ti,ab,kw. | 1960 |
|  |  |  |
| 18 | (Disease$ adj3 rate$).ti,ab,kw. | 27592 |
|  |  |  |
| 19 | (disease$ adj3 frequenc$).ti,ab,kw. | 6000 |
|  |  |  |
| 20 | determinant$.ti,ab,kw. | 213203 |
|  |  |  |
| 21 | (Disease$ adj3 pattern$).ti,ab,kw. | 11292 |
|  |  |  |
| 22 | progress$.ti,ab,kw. | 1002325 |
|  |  |  |
| 23 | long-term.ti,ab,kw. | 711514 |
|  |  |  |
| 24 | trend$.ti,ab,kw. | 348831 |
|  |  |  |
| 25 | epidemiol$.ti,ab,kw. | 362292 |
|  |  |  |
| 26 | exp epidemiology/ | 25196 |
|  |  |  |
| 27 | or/13-26 | 3680156 |
|  |  |  |
| 28 | Developing Countries/ | 71035 |
|  |  |  |
| 29 | (middle income$ adj (countr$ or nation or nations or econom$)).ti,ab,kw. | 12668 |
|  |  |  |
| 30 | (developing adj (countr$ or nation or nations or econom$)).ti,ab,kw. | 55761 |
|  |  |  |
| 31 | ((under developed or underdeveloped) adj (countr$ or nation or nations or econom$)).ti,ab,kw. | 1094 |
|  |  |  |
| 32 | (3rd world adj (countr$ or nation or nations or econom$)).ti,ab,kw. | 58 |
|  |  |  |
| 33 | (third world adj (countr$ or nation or nations or econom$)).ti,ab,kw. | 1066 |
|  |  |  |
| 34 | (LAMI adj (countr$ or nation or nations or econom$)).ti,ab,kw. | 36 |
|  |  |  |
| 35 | (LMIC adj (countr$ or nation or nations or econom$)).ti,ab,kw. | 35 |
|  |  |  |
| 36 | (poor income adj (countr$ or nation or nations or econom$)).ti,ab,kw. | 4 |
|  |  |  |
| 37 | (low$ income adj (countr$ or nation or nations or econom$)).ti,ab,kw. | 5608 |
|  |  |  |
| 38 | (less$ developed adj (countr$ or nation or nations or econom$)).ti,ab,kw. | 1359 |
|  |  |  |
| 39 | (least developed adj (countr$ or nation or nations or econom$)).ti,ab,kw. | 234 |
|  |  |  |
| 40 | asia/ or asia, central/ or asia, northern/ or asia, southeastern/ or asia, western/ or middle east/ or far east/ | 40115 |
|  |  |  |
| 41 | africa/ or africa, northern/ or "africa south of the sahara"/ or africa, central/ or africa, eastern/ or africa, southern/ or africa, western/ | 44932 |
|  |  |  |
| 42 | caribbean region/ or west indies/ | 7370 |
|  |  |  |
| 43 | central america/ or latin america/ or South America/ | 19397 |
|  |  |  |
| 44 | Europe, Eastern/ | 4135 |
|  |  |  |
| 45 | ((South or Southern) adj Asia$).ti,ab,kw. | 7924 |
|  |  |  |
| 46 | ((South East or Southeast$) adj Asia$).ti,ab,kw. | 18489 |
|  |  |  |
| 47 | West$ Asia$.ti,ab,kw. | 750 |
|  |  |  |
| 48 | ((Middle or Far) adj East$).ti,ab,kw. | 14210 |
|  |  |  |
| 49 | Africa$.ti,ab,kw. | 210275 |
|  |  |  |
| 50 | ((Latin or Central or South) adj America$).ti,ab,kw. | 38490 |
|  |  |  |
| 51 | (Caribbean$ or West Indies).ti,ab,kw. | 15538 |
|  |  |  |
| 52 | East$ Europe$.ti,ab,kw. | 7101 |
|  |  |  |
| 53 | (Afghan$ or Angola$ or Armenia$).mp. | 11612 |
|  |  |  |
| 54 | (Bangladesh$ or Benin$ or Bhutan$).mp. | 18488 |
|  |  |  |
| 55 | (Bolivia$ or Burkina Faso$ or Upper Volta$).mp. | 8213 |
|  |  |  |
| 56 | (Burundi$ or Cabo Verd$ or Cape Verd$).mp. | 1515 |
|  |  |  |
| 57 | (Cambodia$ or Cameroon$ or Central African Republic$).mp. | 12805 |
|  |  |  |
| 58 | (Chad$ or Comoros or Comorian or Congo$).mp. | 19605 |
|  |  |  |
| 59 | (Cote d'Ivoire or Ivory Coast or Ivorian$).mp. | 4143 |
|  |  |  |
| 60 | (Djibouti$ or Egypt$ or El Salvador$).mp. | 24517 |
|  |  |  |
| 61 | (Eritrea$ or Ethiopia$ or Gambia$).mp. | 22909 |
|  |  |  |
| 62 | (Georgia$ or Ghana$ or Guatemala$).mp. | 30774 |
|  |  |  |
| 63 | (Guinea$ or Haiti$ or Hondura$).mp. | 166836 |
|  |  |  |
| 64 | (India$ or Indonesia$ or Jordan$).mp. | 215963 |
|  |  |  |
| 65 | (Kenya$ or Kiribati$ or Korea$).mp. | 92192 |
|  |  |  |
| 66 | (Lesotho or Mosotho or Basotho).mp. | 681 |
|  |  |  |
| 67 | (Liberia$ or Madagasca$ or Malagasy).mp. | 7229 |
|  |  |  |
| 68 | (Kosov$ or Kyrgyz$ or Lao$).mp. | 8672 |
|  |  |  |
| 69 | (Malawi$ or Mali or Mali's or Malian$ or Mauritania$).mp. | 11048 |
|  |  |  |
| 70 | (Micronesia$ or Moldov$ or Mongolia$).mp. | 10894 |
|  |  |  |
| 71 | (Morocc$ or Mozambi$ or Myanmar$ or Burma$ or Burmese$).mp. | 17541 |
|  |  |  |
| 72 | (Nepal$ or Nicaragua$ or Niger$).mp. | 61908 |
|  |  |  |
| 73 | (Pakistan$ or PNG$ or Philippines$ or Filipino$).mp. | 36432 |
|  |  |  |
| 74 | (Rwanda$ or Sao Tome$ or Principe$).mp. | 5867 |
|  |  |  |
| 75 | (Senegal$ or Sierra Leone$ or Solomon Island$).mp. | 11381 |
|  |  |  |
| 76 | (Somalia$ or Sudan$ or Sri Lanka$).mp. | 19502 |
|  |  |  |
| 77 | (Swaziland$ or Syria$ or Tajik$).mp. | 13791 |
|  |  |  |
| 78 | (Tanzania$ or Timor$ or Togo$).mp. | 16016 |
|  |  |  |
| 79 | (Tunisia$ or Uganda$ or Ukrain$).mp. | 42880 |
|  |  |  |
| 80 | (Uzbek$ or Vanuatu$ or Vietnam$).mp. | 21183 |
|  |  |  |
| 81 | (West Bank$ or Gaza$ or Palestin$).mp. | 3520 |
|  |  |  |
| 82 | (Yemen$ or Zambia$ or Zimbabwe$).mp. | 14749 |
|  |  |  |
| 83 | (Albania$ or Algeria$ or Argentin$).mp. | 27506 |
|  |  |  |
| 84 | (Samoa$ or Azerbaijan$ or Belarus$).mp. | 5758 |
|  |  |  |
| 85 | (Belize$ or Bosnia$ or Herzegovia$ or Botswana$).mp. | 6453 |
|  |  |  |
| 86 | (Brazil$ or Bulgaria$ or China$ or Chinese$).mp. | 480885 |
|  |  |  |
| 87 | (Colombia$ or Costa Rica$ or Croatia$).mp. | 28230 |
|  |  |  |
| 88 | (Cuba$ or Dominica$ or Ecuador$).mp. | 16463 |
|  |  |  |
| 89 | (Fiji$ or Gabon$ or Grenad$).mp. | 4880 |
|  |  |  |
| 90 | (Guyan$ or Iran$ or Iraq$).mp. | 56182 |
|  |  |  |
| 91 | (Jamaica$ or Kazakh$ or Leban$).mp. | 14275 |
|  |  |  |
| 92 | (Libya$ or Macedonia$ or Malaysia$).mp. | 23392 |
|  |  |  |
| 93 | (Maldiv$ or Marshall Island$ or Mauriti$).mp. | 2285 |
|  |  |  |
| 94 | (Mexic$ or Montenegr$ or Namibia$).mp. | 71841 |
|  |  |  |
| 95 | (Nauru$ or Panama$ or Paraguay$).mp. | 6318 |
|  |  |  |
| 96 | (Peru$ or Romania$ or Russia$).mp. | 88545 |
|  |  |  |
| 97 | (Serbia$ or South Africa$ or Suriname$).mp. | 58637 |
|  |  |  |
| 98 | (Lucia$ or Vincent$ or Thai$).mp. | 40609 |
|  |  |  |
| 99 | (Tonga$ or Turk$ or Turkmen$).mp. | 64124 |
|  |  |  |
| 100 | (Tuvalu$ or Venezuela$).mp. | 9110 |
|  |  |  |
| 101 | or/28-100 | 1968202 |
|  |  |  |
| 102 | 12 and 27 and 101 | 2142 |
|  |  |  |
| 103 | exp animals/ not humans/ | 4511292 |
|  |  |  |
| 104 | 102 not 103 | 2000 |

**Embase(OvidSP) 06/11/18- Embase**1974 to 2018 November 5

|  |  |  |
| --- | --- | --- |
| 1 | exp Osteoarthritis/ | 113504 |
|  |  |  |
| 2 | osteoarthr$.ti,ab,kw. | 89799 |
|  |  |  |
| 3 | OA.ti,ab,kw. | 47010 |
|  |  |  |
| 4 | arthrosis.ti,ab,kw. | 6173 |
|  |  |  |
| 5 | (degenerative adj (arthritis or joint or joints)).ti,ab,kw. | 5181 |
|  |  |  |
| 6 | (knee adj3 (pain or painful)).ti,ab,kw. | 13984 |
|  |  |  |
| 7 | (hip adj3 (pain or painful)).ti,ab,kw. | 7449 |
|  |  |  |
| 8 | (hand$ adj3 (pain or painful)).ti,ab,kw. | 3365 |
|  |  |  |
| 9 | (joint$ adj3 (pain or painful)).ti,ab,kw. | 17690 |
|  |  |  |
| 10 | (finger$ adj3 (pain or painful)).ti,ab,kw. | 826 |
|  |  |  |
| 11 | (thumb$ adj3 (pain or painful)).ti,ab,kw. | 286 |
|  |  |  |
| 12 | or/1-11 | 180095 |
|  |  |  |
| 13 | prevalence/ | 610300 |
|  |  |  |
| 14 | preval$.ti,ab,kw. | 925576 |
|  |  |  |
| 15 | incidence/ | 329702 |
|  |  |  |
| 16 | inciden$.ti,ab,kw. | 1096229 |
|  |  |  |
| 17 | occurence$.ti,ab,kw. | 4997 |
|  |  |  |
| 18 | (Disease$ adj3 rate$).ti,ab,kw. | 42570 |
|  |  |  |
| 19 | (Disease$ adj3 frequenc$).ti,ab,kw. | 8794 |
|  |  |  |
| 20 | determinant$.ti,ab,kw. | 256834 |
|  |  |  |
| 21 | (Disease$ adj3 pattern$).ti,ab,kw. | 15323 |
|  |  |  |
| 22 | progress$.ti,ab,kw. | 1402230 |
|  |  |  |
| 23 | long-term.ti,ab,kw. | 970975 |
|  |  |  |
| 24 | trend$.ti,ab,kw. | 486858 |
|  |  |  |
| 25 | epidemiol$.ti,ab,kw. | 481390 |
|  |  |  |
| 26 | exp epidemiology/ | 2982231 |
|  |  |  |
| 27 | or/13-26 | 6376640 |
|  |  |  |
| 28 | Developing Countries/ | 78424 |
|  |  |  |
| 29 | (middle income$ adj (countr$ or nation or nations or econom$)).ti,ab,kw. | 15235 |
|  |  |  |
| 30 | (developing adj (countr$ or nation or nations or econom$)).ti,ab,kw. | 73280 |
|  |  |  |
| 31 | ((under developed or underdeveloped) adj (countr$ or nation or nations or econom$)).ti,ab,kw. | 1409 |
|  |  |  |
| 32 | (3rd world adj (countr$ or nation or nations or econom$)).ti,ab,kw. | 22 |
|  |  |  |
| 33 | (third world adj (countr$ or nation or nations or econom$)).ti,ab,kw. | 1312 |
|  |  |  |
| 34 | (LAMI adj (countr$ or nation or nations or econom$)).ti,ab,kw. | 46 |
|  |  |  |
| 35 | (LMIC adj (countr$ or nation or nations or econom$)).ti,ab,kw. | 65 |
|  |  |  |
| 36 | (poor income adj (countr$ or nation or nations or econom$)).ti,ab,kw. | 4 |
|  |  |  |
| 37 | (low$ income adj (countr$ or nation or nations or econom$)).ti,ab,kw. | 7434 |
|  |  |  |
| 38 | (less$ developed adj (countr$ or nation or nations or econom$)).ti,ab,kw. | 1492 |
|  |  |  |
| 39 | (least developed adj (countr$ or nation or nations or econom$)).ti,ab,kw. | 272 |
|  |  |  |
| 40 | asia/ or asia, central/ or asia, northern/ or asia, southeastern/ or asia, western/ or middle east/ or far east/ | 82677 |
|  |  |  |
| 41 | africa/ or africa, northern/ or "africa south of the sahara"/ or africa, central/ or africa, eastern/ or africa, southern/ or africa, western/ | 60363 |
|  |  |  |
| 42 | caribbean region/ or west indies/ | 5137 |
|  |  |  |
| 43 | central america/ or latin america/ or South America/ | 30130 |
|  |  |  |
| 44 | Europe, Eastern/ | 5952 |
|  |  |  |
| 45 | ((South or Southern) adj Asia$).ti,ab,kw. | 11059 |
|  |  |  |
| 46 | ((South East or Southeast$) adj Asia$).ti,ab,kw. | 18289 |
|  |  |  |
| 47 | West$ Asia$.ti,ab,kw. | 875 |
|  |  |  |
| 48 | ((Middle or Far) adj East$).ti,ab,kw. | 18499 |
|  |  |  |
| 49 | Africa$.ti,ab,kw. | 255243 |
|  |  |  |
| 50 | ((Latin or Central or South) adj America$).ti,ab,kw. | 46828 |
|  |  |  |
| 51 | (Caribbean$ or West Indies).ti,ab,kw. | 17324 |
|  |  |  |
| 52 | East$ Europe$.ti,ab,kw. | 9554 |
|  |  |  |
| 53 | (Afghan$ or Angola$ or Armenia$).mp. | 12878 |
|  |  |  |
| 54 | (Bangladesh$ or Benin$ or Bhutan$).mp. | 24690 |
|  |  |  |
| 55 | (Bolivia$ or Burkina Faso$ or Upper Volta$).mp. | 9472 |
|  |  |  |
| 56 | (Burundi$ or Cabo Verd$ or Cape Verd$).mp. | 1615 |
|  |  |  |
| 57 | (Cambodia$ or Cameroon$ or Central African Republic$).mp. | 14933 |
|  |  |  |
| 58 | (Chad$ or Comoros or Comorian or Congo$).mp. | 27690 |
|  |  |  |
| 59 | (Cote d'Ivoire or Ivory Coast or Ivorian$).mp. | 4652 |
|  |  |  |
| 60 | (Djibouti$ or Egypt$ or El Salvador$).mp. | 33835 |
|  |  |  |
| 61 | (Eritrea$ or Ethiopia$ or Gambia$).mp. | 25356 |
|  |  |  |
| 62 | (Georgia$ or Ghana$ or Guatemala$).mp. | 28044 |
|  |  |  |
| 63 | (Guinea$ or Haiti$ or Hondura$).mp. | 138893 |
|  |  |  |
| 64 | (India$ or Indonesia$ or Jordan$).mp. | 293986 |
|  |  |  |
| 65 | (Kenya$ or Kiribati$ or Korea$).mp. | 138469 |
|  |  |  |
| 66 | (Lesotho or Mosotho or Basotho).mp. | 793 |
|  |  |  |
| 67 | (Liberia$ or Madagasca$ or Malagasy).mp. | 7827 |
|  |  |  |
| 68 | (Kosov$ or Kyrgyz$ or Lao$).mp. | 7900 |
|  |  |  |
| 69 | (Malawi$ or Mali or Mali's or Malian$ or Mauritania$).mp. | 13175 |
|  |  |  |
| 70 | (Micronesia$ or Moldov$ or Mongolia$).mp. | 12118 |
|  |  |  |
| 71 | (Morocc$ or Mozambi$ or Myanmar$ or Burma$ or Burmese$).mp. | 21197 |
|  |  |  |
| 72 | (Nepal$ or Nicaragua$ or Niger$).mp. | 77971 |
|  |  |  |
| 73 | (Pakistan$ or PNG$ or Philippines$ or Filipino$).mp. | 48644 |
|  |  |  |
| 74 | (Rwanda$ or Sao Tome$ or Principe$).mp. | 6463 |
|  |  |  |
| 75 | (Senegal$ or Sierra Leone$ or Solomon Island$).mp. | 12643 |
|  |  |  |
| 76 | (Somalia$ or Sudan$ or Sri Lanka$).mp. | 22187 |
|  |  |  |
| 77 | (Swaziland$ or Syria$ or Tajik$).mp. | 19029 |
|  |  |  |
| 78 | (Tanzania$ or Timor$ or Togo$).mp. | 18097 |
|  |  |  |
| 79 | (Tunisia$ or Uganda$ or Ukrain$).mp. | 47767 |
|  |  |  |
| 80 | (Uzbek$ or Vanuatu$ or Vietnam$).mp. | 20586 |
|  |  |  |
| 81 | (West Bank$ or Gaza$ or Palestin$).mp. | 4108 |
|  |  |  |
| 82 | (Yemen$ or Zambia$ or Zimbabwe$).mp. | 15690 |
|  |  |  |
| 83 | (Albania$ or Algeria$ or Argentin$).mp. | 36663 |
|  |  |  |
| 84 | (Samoa$ or Azerbaijan$ or Belarus$).mp. | 6732 |
|  |  |  |
| 85 | (Belize$ or Bosnia$ or Herzegovia$ or Botswana$).mp. | 7645 |
|  |  |  |
| 86 | (Brazil$ or Bulgaria$ or China$ or Chinese$).mp. | 604818 |
|  |  |  |
| 87 | (Colombia$ or Costa Rica$ or Croatia$).mp. | 39483 |
|  |  |  |
| 88 | (Cuba$ or Dominica$ or Ecuador$).mp. | 19687 |
|  |  |  |
| 89 | (Fiji$ or Gabon$ or Grenad$).mp. | 5571 |
|  |  |  |
| 90 | (Guyan$ or Iran$ or Iraq$).mp. | 77772 |
|  |  |  |
| 91 | (Jamaica$ or Kazakh$ or Leban$).mp. | 16016 |
|  |  |  |
| 92 | (Libya$ or Macedonia$ or Malaysia$).mp. | 30464 |
|  |  |  |
| 93 | (Maldiv$ or Marshall Island$ or Mauriti$).mp. | 2856 |
|  |  |  |
| 94 | (Mexic$ or Montenegr$ or Namibia$).mp. | 80216 |
|  |  |  |
| 95 | (Nauru$ or Panama$ or Paraguay$).mp. | 6437 |
|  |  |  |
| 96 | (Peru$ or Romania$ or Russia$).mp. | 136543 |
|  |  |  |
| 97 | (Serbia$ or South Africa$ or Suriname$).mp. | 67543 |
|  |  |  |
| 98 | (Lucia$ or Vincent$ or Thai$).mp. | 50793 |
|  |  |  |
| 99 | (Tonga$ or Turk$ or Turkmen$).mp. | 85133 |
|  |  |  |
| 100 | (Tuvalu$ or Venezuela$).mp. | 9887 |
|  |  |  |
| 101 | or/28-100 | 2439211 |
|  |  |  |
| 102 | 12 and 27 and 101 | 5163 |
|  |  |  |
| 103 | exp animals/ not humans/ | 11617879 |
|  |  |  |
| 104 | 102 not 103 | 1999 |
